# Mood computational mechanisms underlying increased risk behavior in adolescent suicidal patients

**DOI:** 10.7554/eLife.108002

**Published:** 2026-08-07

**Authors:** Zhihao Wang, Tian Nan, Fengmei Lu, Yu Yue, Xiao Cai, Zongling He, Yuejia Luo, Ting Wang, Bastien Blain

**Affiliations:** 1 Center for Neurocognition and Social Behavior, Institute of Artificial Intelligence, Shenzhen University of Advanced Technology Shenzhen China; 2 CNRS - Centre d'Economie de la Sorbonne, Panthéon-Sorbonne University Paris France; 3 Beijing Key Laboratory of Applied Experimental Psychology, National Demonstration Center for Experimental Psychology Education (BNU), Faculty of Psychology, Beijing Normal University Beijing China; 4 The Clinical Hospital of Chengdu Brain Science Institute, MOE Key Lab for Neuroinformation, University of Electronic Science and Technology of China Chengdu China; 5 China-Cuba Belt and Road Joint Laboratory on Neurotechnology and Brain-Apparatus Communication, University of Electronic Science and Technology of China Chengdu China; 6 Institute for Brain Research and Rehabilitation, South China Normal University Guangzhou China; 7 Department of Experimental Psychology, University College London London United Kingdom; University College London United Kingdom; University College London United Kingdom

**Keywords:** computational psychiatry, suicide, risk decision making, affective disorder, adolescence, subjective wellbeing, Human

## Abstract

Suicidal thoughts and behaviors (STB) are among the leading causes of death worldwide. Although previous research has consistently documented elevated risk-taking in individuals with STB and identified mood disturbances as central features of suicidality, the precise cognitive and affective computational mechanisms underlying this increased risky behavior remain poorly understood. Here, 83 adolescent inpatients with affective disorders—including 58 patients with STB (S^+^) and 25 without STB (S^−^)—and 118 age- and sex-matched healthy controls (HC) completed a decision-making task involving choices between certain and gamble options, alongside momentary mood ratings. Behavioral analyses showed that S^+^ exhibited greater risk-taking than both S^−^ and HC. Computational modeling of choice behavior using a prospect-theory framework augmented with value-insensitive approach–avoidance parameters indicated that this increase in risky behavior was specifically driven by an elevated approach parameter in S^+^. In addition, mood-model analyses revealed reduced sensitivity to certain rewards in S^+^ relative to S^−^ and HC. Importantly, these computational signatures predicted suicidal symptom severity and showed generalizability in an independent general-population sample (*n* = 747). In S^+^, lower mood sensitivity to certain rewards was associated with greater gambling, providing a computational affective account of increased risk-taking in STB. These findings remained robust after adjusting for demographic, clinical, and medication-related variables. Overall, our study identifies cognitive and affective computational mechanisms contributing to elevated risk-taking in STB and highlights their potential relevance for the early identification and prevention of suicidality.

## Introduction

Every 40 s, a life is lost due to suicide (WHO, 2014). Suicidal thoughts and behaviors (STB) are one of the leading causes of death worldwide that have devastating impacts on individuals, families, and societies. STB occurs from adolescence (Hawton et al., 2012), especially in the context of mood disorders, for example, major depressive disorder (MDD), anxiety disorder (AD), and bipolar disorder (BD) (Guze and Robins, 1970). Despite the progress made during the last 50 years in identifying risk factors (Franklin et al., 2017) and developing preventive strategies (King et al., 2018), the death rate from STB has not declined (Schmaal et al., 2020). The limited comprehension of cognitive and affective mechanisms creates a substantial gap in pinpointing targets for early prediction, screening, detection, and intervention in cases of suicidal thoughts. Understanding what is impaired in STB patients’ decision process would be key to preventing STB, for example through cognitive behavioral therapy (da Silva et al., 2018; Oldershaw et al., 2012).

Although meta-analyses have shown increased risk behavior in patients with STB across different risk domains (for a short summary, see Appendix 1—table 1; Sastre-Buades et al., 2021; Perrain et al., 2021; Richard-Devantoy et al., 2014), the underlying cognitive computational mechanism is still unknown. Specifically, some studies found heightened loss aversion in STB in the context of the balloon analog risk task and the gambling task (Liu et al., 2022; Baek et al., 2017), while others observed the opposite pattern using the Iowa gambling task (Alacreu-Crespo et al., 2020). Although all these results aligned with their hypotheses (for a short summary, see Appendix 1—table 1), this contradictory evidence may originate from the use of underspecified models. A growing literature indeed shows that risky behavior can be far better explained after adding value-insensitive approach and avoidance components to prospect theory (Rutledge et al., 2015; Rutledge et al., 2016b), that is by including a decision bias in favor of the highest gain (approach) and another decision bias against the lowest loss (avoidance), above and beyond options value difference. This class of models highlights the important role of value-insensitive motivational components in decision making in addition to risk attitude-driven valuation (e.g., loss/risk aversion) (Corr and McNaughton, 2012). Importantly, STB has been proposed in theoretical work to result from an abnormal motivational system (Dombrovski and Hallquist, 2022; Karvelis and Diaconescu, 2022; Millner et al., 2019; Dombrovski and Hallquist, 2017), but no direct evidence supports such proposals. Therefore, investigating motivational components may facilitate understanding why STB is associated with increased risk-taking behavior. We therefore hypothesized that heightened approach motivation, or weakened avoidance motivation, would account for increased risk behavior in STB.

While suicide is a decision process per se, atypical mood dynamics have been thought to be at the core of STB. Contemporary theories of suicide converge on the idea that STB is initially caused by low mood experience. The interpersonal theory of suicide proposes that suicidal desire arises when people simultaneously feel socially disconnected (thwarted belongingness) and like a burden on others (perceived burdensomeness), experiences that are tightly linked to chronically low mood (Van Orden et al., 2010). The motivational–volitional model (O’Connor et al., 2016) and the three-step theory (Millner et al., 2020; Rutledge et al., 2014) similarly emphasize that when negative mood and feelings of defeat or entrapment are experienced as inescapable, they can give rise to suicidal ideation, and that the progression from ideation to suicide attempts depends on additional factors such as reduced fear of death, increased pain tolerance, and a tendency to act impulsively under intense affect. Some official organizations, for example, National Institute of Mental Health, have also listed mood problems as warning signals (Franklin et al., 2017). Interestingly, within the framework of decision making under uncertainty, gambling on lotteries with a revealed outcome has been found to induce high mood variance (Rutledge et al., 2014), providing an opportunity to assess the relationship between deficient mood and increased gambling decisions in STB. Specifically, in a gambling task with momentary mood ratings (also referred to as happiness or subjective well-being), where participants were asked to make decisions between certain vs. gamble options (2 possible outcomes, 50% probability for each), Rutledge et al., 2014 found that mood was sensitive to certain rewards (CR), reward expectation (EV), and reward prediction error (RPE; the difference between experienced and expected outcome) (Rutledge et al., 2014). Although mood is thought to persist for hours, days, or even weeks (Kao et al., 2023; Emanuel and Eldar, 2023; Eldar et al., 2016; Schiller et al., 2024), momentary mood, measured over the timescale in the laboratory setting, represents the accumulation of the impact of multiple events at the scale of minutes (Kao et al., 2023; Eldar et al., 2016; Rutledge et al., 2016a; Blain and Rutledge, 2020; Rutledge et al., 2014; Rutledge et al., 2017; Csukly et al., 2023). Momentary mood external validity is demonstrated, for example, through its association with depression symptoms (Rutledge et al., 2017). Mood is different from emotions, which reflect immediate affective reactivity and are more transient (e.g., from surprise to fear) (Emanuel and Eldar, 2023; Eldar et al., 2016; Schiller et al., 2024; Wang et al., 2023). Here, we investigated which mood computational components (among CR, EV, and RPE) are associated with STB. We expect the mood response to gambling-related quantities (EV and RPE) to be higher in STB compared to the control groups. In contrast, riskier decisions may result from aversion to CR in STB. Therefore, another possibility is that lower mood sensitivity to CR would relate to increased risk behavior in STB.

To summarize, the aim of this study is to examine cognitive and affective computational mechanisms underlying increased risk behavior in adolescent patients with STB, as the adolescent period might provide a developmental window for opportunities for early intervention (Hawton et al., 2012). This study aligns with the principles of Computational Psychiatry (Huys et al., 2016), which assumes that psychiatric symptoms arise from alterations in cognitive and affective computations. The ultimate aim for this field is to uncover ‘computational phenotypes’—distinct patterns of computational dysfunctions—potentially enabling targeted treatments, improved outcome predictions, and more precise diagnostic frameworks. Specifically, we employed a gambling task with momentary mood ratings to assess risk behavior and track mood fluctuations in response to various events. We applied computational models of risky decision making and momentary mood to dissect cognitive and affective processes contributing to heightened risky behavior in patients with STB. Regarding choices, we hypothesized heightened approach motivation, or weakened avoidance motivation, in STB, which would account for increased risk behavior. Regarding mood dynamics, we hypothesized that greater mood sensitivity to gambling-related variables (i.e., RPE and EV), or reduced mood sensitivity to CR, would explain increased risk behavior in STB.

## Methods

### Participants

We recruited 95 adolescent patients with mood disorder from the Clinical Hospital of Chengdu Brain Science Institute, University of Electronic Science and Technology of China (The Mental Health Center of Chengdu, Sichuan, China). According to medical records and information from family and friends by the researcher (T.N.) and psychiatrists (F.L., Y.Y., X.C., and Z.H.), patients with suicidal thoughts and behaviors were categorized as the suicidal group (S^+^), while patients without suicidal thoughts and behaviors were identified as the control group (S^−^). The definition for suicidal thoughts in this study was active thoughts of suicide, that is, wishing to die and having some intention to do so (see Appendix 1 for details). This grouping operation was consistent with previous suicidal-related literature (Glenn et al., 2019; Glenn et al., 2017; Millner et al., 2019; Miller et al., 2024; Eisenlohr-Moul et al., 2018; Miller et al., 2017), reflecting the general tendency for suicidal risks among adolescents. As baseline control, we also recruited 124 sex- and age-matched healthy adolescents (HC). We assert that all procedures contributing to this work comply with the ethical standards of the ethical committee of The Clinical Hospital of Chengdu Brain Science Institute, University of Electronic Science and Technology of China (number: 2022 [Rutledge et al., 2016a]) on human experimentation and with the Helsinki Declaration of 1975, as revised in 2008. All procedures involving human subjects/patients were approved by the ethical committee of The Clinical Hospital of Chengdu Brain Science Institute, University of Electronic Science and Technology of China (number: 2022 [Rutledge et al., 2016a]). Informed written consent was obtained. Patients were included if they met the following criteria: (1) both the researcher and psychiatrists agreed on their group classification; (2) they had a current diagnosis of major depressive disorder (MDD; unipolar depression), generalized anxiety disorder (GAD), or bipolar disorder with depressive episodes (BD), confirmed by two experienced psychiatrists using the Structured Clinical Interview for DSM-IV-TR-Patient Edition (SCID-P, 2/2001 revision; see Appendix 1 for details); (3) they were between 10 and 19 years of age; (4) they had no organic brain disorders, intellectual disability, or head trauma; (5) they had no history of substance abuse; (6) they had no experience of electroconvulsive therapy. In addition, participants were excluded if they failed more than 1/4 of the catch trials. The final sample consisted of 25 patients for S^−^, 58 patients for S^+^, and 118 HC participants. See Table 1, Appendix 1—table 2 for demographic, clinical, and psychological information. The validation dataset was from our previous online study, with 747 general participants completing the same task and numerous anxiety/depression-related questionnaires for different purposes. See for demographic and psychological details.

**Table 1.** Demographics, clinical, psychological characteristics of patients with and without suicidal thoughts and behaviors.

	Group			Group contrast		S^+^ vs. S^−^	
	HC (*n* = 118)	S^−^ (*n* = 25)	S^+^ (*n* = 58)	*F*/*χ*^2^	p	*t*/*χ*^2^	p
Sex (female/male)	75/43	16/9	41/17	0.912	0.634	0.363	0.547
Age	15.31 ± 2.15	15.68 ± 1.75	14.83 ± 1.80	1.868	0.157	**1.997**	**0.049**
BSI-C	1.29 ± 3.62	2.84 ± 2.66	18.02 ± 7.56	224.230	<0.001	–9.754	<0.001
BSI-W	3.58 ± 6.60	4.04 ± 3.22	27.98 ± 6.02	326.242	<0.001	–18.723	<0.001
CTQ	13.98 ± 11.29	22.64 ± 12.34	33.00 ± 17.03	40.023	<0.001	–2.743	0.008
ERQ-R	14.77 ± 4.38	13.08 ± 6.34	8.48 ± 5.39	31.317	<0.001	3.376	0.001
ERQ-S	6.86 ± 3.64	8.80 ± 3.77	10.79 ± 3.79	22.322	<0.001	–2.200	0.031
Suicidal attempts history (yes)	---	---	29	---	---	---	---
Illness duration (months)	---	31.76 ± 18.80	31.38 ± 18.70	---	---	0.085	0.933
Family history (yes)	---	2	10	---	---	1.206	0.272
Current diagnosis (GAD/MDD/BD)	---	24/55/9	10/17/6	---	---	1.790	0.409
Medication (yes)	---	25	57	---	---	0.436	0.509
SSRI	---	16	39	---	---	0.082	0.775
SNRI	---	0	2	---	---	0.883	0.347
Trazodone	---	6	16	---	---	0.115	0.734
Antipsychotics	---	14	32	---	---	0.005	0.945
BZDs	---	20	45	---	---	0.060	0.807
Other anxiolytics	---	12	13	---	---	**5.434**	**0.020**
Mood stabilizer	---	13	18	---	---	3.282	0.070
TAI	43.49 ± 8.54	50.38 ± 12.19	65.36 ± 7.62	108.863	<0.001	–6.276	<0.001
PSWQ	44.75 ± 10.94	50.67 ± 15.17	68.56 ± 9.58	80.213	<0.001	–5.990	<0.001
BDI	9.45 ± 9.43	18.62 ± 15.11	38.30 ± 9.67	129.516	<0.001	–6.573	<0.001
CESD	32.96 ± 11.09	42.86 ± 15.66	62.52 ± 9.99	118.084	<0.001	–6.347	<0.001

Note: For anxiety/depression-related questionnaires (TAI, PSWQ, BDI, and CESD), due to time limitation, data from eight participants in the S^+^ group and four participants in the S^−^ group was not collected. Bold values indicate an unexpected statistically significant difference. Abbreviations: HC, healthy control; S^−^, patients without suicidal thoughts and behavior; S^+^, patients with suicidal thoughts and behavior; BSI-C, Beck Scale for Suicidal Ideation at the current time; BSI-W, Beck Scale for Suicidal Ideation at the worst time; CTQ, Childhood Trauma Questionnaire; ERQ-R, Emotion Regulation Questionnaire-Reappraisal; ERQ-S, Emotion Regulation Questionnaire-Suppression; AD, anxiety disorders; MDD, major depressive disorders; BD, bipolar disorders; SSRI, Selective Serotonin Reuptake Inhibitor; SNRI, serotonin-norepinephrine reuptake inhibitors; BZDs, Benzodiazepines; TAI, Trait Anxiety Inventory; PSWQ, Penn State Worry Questionnaire; BDI, Beck Depression Inventory; CESD, Center for Epidemiologic Studies Depression Scale.

### Self-reported questionnaires

Participants completed a set of Chinese-version suicidal-, emotion regulation-, and depression/anxiety-related questionnaires. These measurements included the Beck Scale for Suicidal Ideation at the current time (BSI-C, 19 items) and at the worst time (BSI-W, 19 items) (Zhang and Brown, 2007), the Childhood Trauma Questionnaire (CTQ, 28 items) (Zhao et al., 2005), Emotion Regulation Questionnaire-Reappraisal (ERQ-R, 6 items), and Suppression (ERQ-S, 4 items) (Zhu et al., 2008). In addition, as patients were available only for a limited duration, anxiety/depression-related scales from only 50 participants in the S^+^ group and only 21 participants in the S^−^ group were collected. Specifically, patients filled the Trait subscale of the State-Trait Anxiety Inventory (TAI; 20 items) (Shek, 1988), the Penn State Worry Questionnaire (PSWQ; 16 items), the Beck Depression Inventory (BDI; 21 items) (Shek, 1990), and the Center for Epidemiologic Studies Depression Scale (CESD; 20 items) (Jiang et al., 2019).

### Experimental procedure

**Figure 1.**
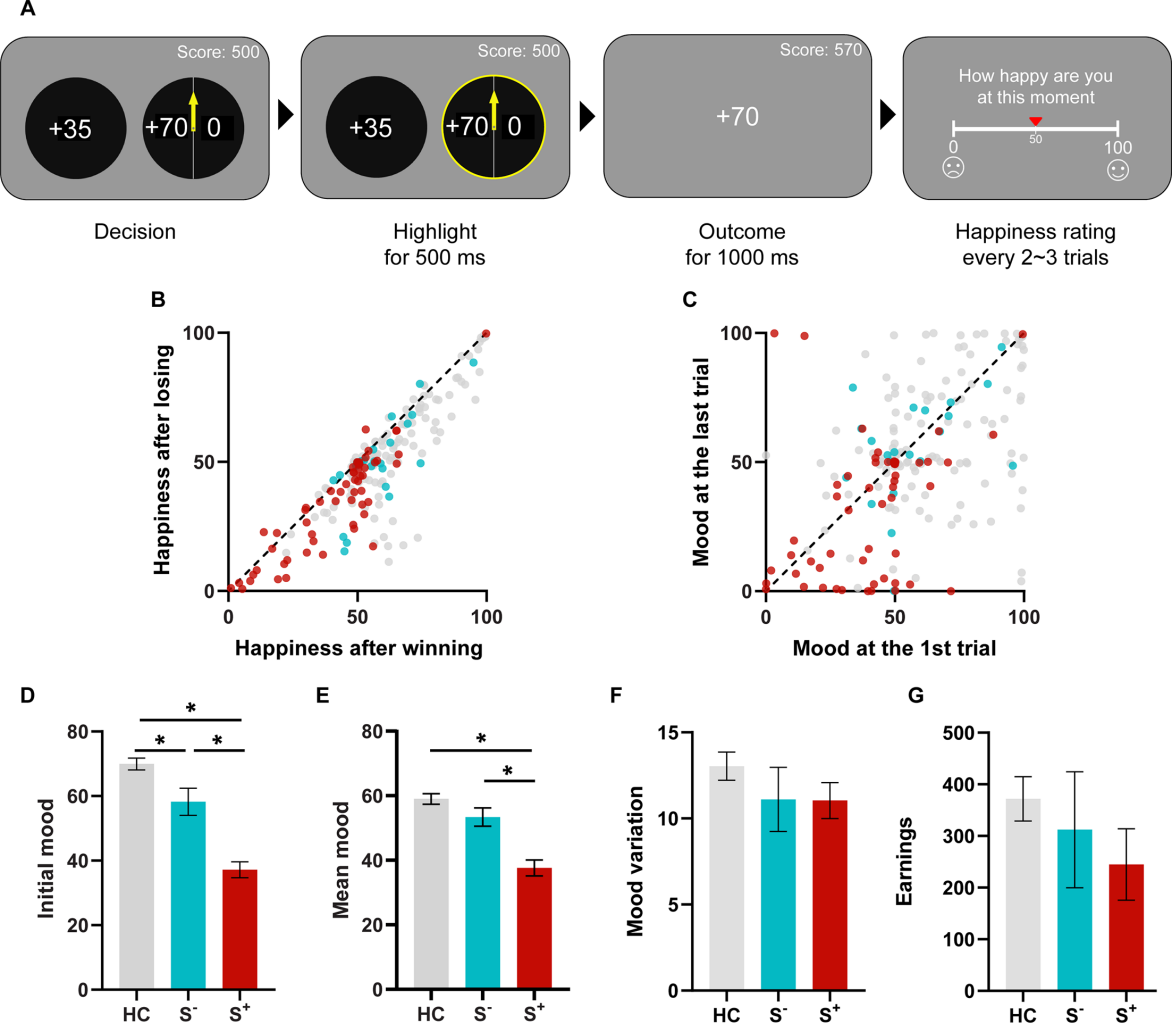
Task design, outcome and time effects on mood, and group differences in mood. (**A**) Gambling task with mood ratings. On each trial, participants were asked to choose between a certain option and a gambling option (self-paced). Once selected, the chosen option was highlighted in yellow for 500 ms. Then the corresponding outcome was displayed in the center of the screen for 1000 ms. The cumulated score was always shown in the right-upper corner. Every two to three trials, participants were asked to complete a self-paced rating of their happiness, answering the question ‘How happy are you at the moment’ on a slider from 0 (very unhappy) to 100 (very happy). (**B**) Patients and healthy controls felt happier after winning than losing. (**C**) Mood drifted over time. (**D**) Group difference in mood before the task shows weakened mood in S^+^. (**E**) Group difference in average mood displays lower mood experience in S^+^. (**F**) Mood variance was similar for all three groups, as indexed by the standard deviation of happiness ratings across the task. (**G**) Each group earned about the same amount of points by the end of the task. Abbreviations: HC, healthy control; S^−^, patients without suicidal thoughts and behavior; S^+^, patients with suicidal thoughts and behavior; *p < 0.05. Error bars correspond to the standard error.

Participants were asked to make a choice between a certain option and a gamble (50% probability for each outcome) to maximize their points and to rate their momentary moods (Rutledge et al., 2015; Rutledge et al., 2014). Before performing the task, participants were asked to rate their current happiness that we consider as their initial mood. At the beginning of the task, participants were endowed with 500 points. Each trial started with two options (a gamble option and a certain option) which were presented randomly on each side (Figure 1A). Upon response, the chosen option was highlighted in yellow for 0.5 s. Note that Rutledge et al., 2014 displayed the chosen option for about 6 s (Rutledge et al., 2014), a delay we shortened for the sake of time. Then the corresponding outcome at the screen center was presented for 1 s, followed by a fixation cross with a random duration (0.6–1.4 s). If the gamble was chosen, participants had equal probability to obtain each outcome. The obtained outcome was added to their total score, which was presented at the top-right corner. Every two to three trials, participants rated their mood (how happy are you at this moment?) from 0 (very unhappy) to 100 (very happy) by moving a slider anchored at midpoint (i.e., 50). Upon identifying their current mood, a fixation cross was presented with a random duration (0.6–1.4 s). This task consisted of 90 randomly presented trials, including 30 mixed trials, 30 gain trials, and 30 loss trials. The numbers of choice trials and mood ratings were comparable to those in prior computational modeling studies (Blain and Rutledge, 2020; Rutledge et al., 2014). In mixed trials, participants made a choice between a certain amount 0 and a gamble with a gain amount {40, 45, or 75} and a loss amount determined by a multiplier {0.2, 0.34, 0.5, 0.64, 0.77, 0.89, 1, 1.1, 1.35, or 2} on the gain amount. For example, with a gain amount of 40 and a multiplier of 2 for the loss (2 times 40 = 80), participants chose between a certain option of 0 and a gambling option, which offered a 50% chance to win 40 and a 50% chance to lose 80. These trials are therefore particularly suited to measuring loss aversion. In gain trials, there was a certain gain amount {35, 45, or 55} and a gamble with 0 and a gain amount determined by a multiplier {1.68, 1.82, 2, 2.22, 2.48, 2.8, 3.16, 3.6, 4.2, or 5} on the certain gain amount. In loss trials, there were a certain loss amount {−35,–45, or –55} and a gamble with 0 and a loss amount determined by a multiplier {1.68, 1.82, 2, 2.22, 2.48, 2.8, 3.16, 3.6, 4.2, or 5} on the certain loss amount. We also added an extra four trials in the entire task for attentional checks. For example, participants were asked to make a choice between a certain gain of 20 and a gambling amount of 35/55, where the correct response for this trial was the gambling choice (as the worst lottery outcome was higher than the certain reward). All experimental procedures were programmed using Psychopy3 (2021.2.3).

### Choice computational models

In line with previous studies (Rutledge et al., 2015; Rutledge et al., 2016b), our choice model space included expected value model (cM1), prospect theory model (cM2) (Kahneman and Tversky, 1979), and approach–avoidance prospect theory model (cM3) (Rutledge et al., 2015). For cM2 (Equations 1–4), there were three parameters, including risk aversion (*α*, range: [0.3, 1.3]), loss aversion (λ: [0.5, 5]), and inverse temperature (*µ*: [0, 10]).

(1) $$\displaystyle U_{gamble}=0.5\left (V_{gain}\right)^{\alpha }-{\, }0.5\mathrm{\lambda }\left (-V_{loss}\right)^{\alpha }$$

(2) $$\displaystyle U_{certain}=\left (V_{certain}\right)^{\alpha }{\,}if\mathrm{\ }V_{certain}\geq 0$$

(3) $$\displaystyle U_{certain}=-\mathrm{\lambda }\left (-V_{certain}\right)^{\alpha }{\, }if{\, }V_{certain}< 0$$

(4) $$\displaystyle P_{gamble}={\, }\frac{1}{1+e^{-\mu \left (U_{gamble}-U_{certain}\right)}}$$

where *V_gain_* and *V_loss_* are the objective gain and loss from a gamble, respectively. Please note that *V_gain_* is 0 in loss trials and *V_loss_* is 0 in gain trials. *V_certain_* is the objective value for the certain option. *U_gamble_* and *U_certain_* denote the subjective utilities of the gamble and the certain option, respectively. Choice probability for gamble (*P_gamble_*) is determined by the softmax rule. Building on cM2, cM3 decomposes the decision process into risk-attitude-driven valuation (e.g., loss and risk aversion) and value-insensitive motivational components (Equations 1–3; 5–7). That is, choice probability for *P_gamble_* in cM3 is jointly determined by the softmax rule and approach/avoidance parameters ($\beta _{gain}$: [–1, 1], $\beta _{loss}$: [–1, 1]). Approach/avoidance parameters are not applied in mixed trials. Please note that a higher gambling rate does not imply a change in risk attitude per se: it can arise from an increased value-insensitive approach bias even when risk-attitude parameters are comparable between groups. Risk attitude is indeed conceptualized in economics as the curvature of the utility function (i.e., the subjective value) of the objective outcomes, with concave curves associated with risk aversion, and convex curves associated with risk seeking (Sokol-Hessner and Rutledge, 2019; Rutledge et al., 2015). By contrast, the approach or avoidance bias applies to all the value. A possible interpretation of the approach bias is that participants approach the option with the highest possible gain (the lottery) in the gain frame; the avoidance bias would then reflect a tendency to systematically avoid the highest potential losses (the lottery) in the loss frame.

(5) $$\displaystyle P_{gamble}={\, }\frac{1-\beta _{val}}{1+e^{-\mu \left (U_{gamble}-U_{certain}\right)}}+\beta _{val}{\, \, }if{\, }\beta _{val}\geq 0$$

(6) $$\displaystyle P_{gamble}=\frac{1+\beta _{val}}{1+e^{-\mu \left (U_{gamble}-U_{certain}\right)}}{\, \, }if{\, }\beta _{val}< 0$$

(7) $$\displaystyle \beta _{val}\left \{\begin{array}{c}\beta _{gain},\, \, gain\, trials,\\\beta _{loss},\, \, loss\, trials.\end{array}\right.$$

### Mood computational models

To quantify how different events impacted participants’ momentary mood during the gambling task, we conducted a stage-wise model construction procedure (Wang et al., 2025). That is, we added or removed each component to the model progressively, based on the best model from the previous stage. In Stage 1, we fit the classic model assuming that momentary mood depends on the recency-weighted average of the chosen certain reward (CR), expected value of the chosen gamble (EV), and reward prediction error (RPE; mM1; Equation 8). RPE was defined as the difference between the obtained and expected value.

(8) $$\displaystyle  \mathrm{H}\mathrm{a}\mathrm{p}\mathrm{p}\mathrm{i}\mathrm{n}\mathrm{e}\mathrm{s}\mathrm{s}\left (t\right)=\, \beta _{0}+\beta _{CR}\sum \limits_{j=1}^{t}\gamma ^{t-j}CR_{j}+\beta _{EV}\sum \limits_{j=1}^{t}\gamma ^{t-j}EV_{j}+\beta _{RPE}\sum \limits_{j=1}^{t}\gamma ^{t-j}RPE_{j}$$

Here, *t* and *j* are trial numbers, $\beta _{0}$ is a baseline mood parameter, other weights $\beta $ capture the influence of different event types, $\mathrm{\gamma }\in $ [0,1] is a decay parameter representing how many previous trials influence happiness. *CR_j_* is the CR if the certain option was chosen on trial *j*; otherwise, *CR_j_* is 0. *EV_j_* is the EV and *RPE_j_* is the RPE on trial *j* if the gamble was chosen. If the certain option was chosen, then *EV_j_* = 0 and *RPE_j_* = 0.

To check that mood ratings are best explained by a shared forgetting factor (i.e., the recency-weighted history of different event types), we compared a model with a single decay parameter to an alternative model, including forgetting factors for each event type, e.g., different decay parameters for CR, EV, and RPE (mM2; Equation 9).

(9) $$\displaystyle  \mathrm{H}\mathrm{a}\mathrm{p}\mathrm{p}\mathrm{i}\mathrm{n}\mathrm{e}\mathrm{s}\mathrm{s}\left (t\right)=\, \beta _{0}+\beta _{CR}\sum \limits_{j=1}^{t}\gamma _{CR}^{t-j}CR_{j}+\beta _{EV}\sum \limits_{j=1}^{t}\gamma _{EV}^{t-j}EV_{j}+\beta _{RPE}\sum \limits_{j=1}^{t}\gamma _{RPE}^{t-j}RPE_{j}$$

In Stage 2, to identify whether mood can be better explained by RPE, we fit an alternative model in which mood ratings are explained by the recency-weighted average of the certain reward (CR) and the gamble reward (GR; mM3; Equation 10), a simple model providing a mood sensitivity parameter for certain rewards and gamble rewards. We also fit a model with two forgetting factors, one for CR and one for GR (mM4; Equation 11).

(10) $$\displaystyle  \mathrm{H}\mathrm{a}\mathrm{p}\mathrm{p}\mathrm{i}\mathrm{n}\mathrm{e}\mathrm{s}\mathrm{s}\left (t\right)=\, \beta _{0}+\beta _{CR}\sum \limits_{j=1}^{t}\gamma ^{t-j}CR_{j}+\beta _{GR}\sum \limits_{j=1}^{t}\gamma ^{t-j}GR_{j}$$

(11) $$\displaystyle  \mathrm{H}\mathrm{a}\mathrm{p}\mathrm{p}\mathrm{i}\mathrm{n}\mathrm{e}\mathrm{s}\mathrm{s}\left (t\right)=\, \beta _{0}+\beta _{CR}\sum \limits_{j=1}^{t}\gamma _{CR}^{t-j}CR_{j}+\beta _{GR}\sum \limits_{j=1}^{t}\gamma _{GR}^{t-j}GR_{j}$$

In Stage 3, to check whether mood data can be better explained by a single event (CR or GR), we compared a CR-mood model (mM5) and a GR-mood model (mM6).

### Model fitting and comparison

We fit model parameters by using the method of maximum likelihood estimation (MLE) with the fmincon function of MATLAB (version R2015a) at the individual level. To avoid local minima, we ran this optimization function with random starting locations 50 times. Bayesian information criteria (BIC) were used to compare model fits.

### Replication of suicidal-related results in an independent dataset (*n* = 747)

We next verified our results in an independent dataset, including the same task and BDI questionnaire in 747 general participants (500 females; age: 20.90 ± 2.41) (Wang et al., 2025). One item in BDI involves the measurement of STB. In item 9 of BDI, participants chose one option that describes them best: Option 1, ‘I don't have any thoughts of killing myself.’; Option 2, ‘I have thoughts of killing myself, but I would not carry them out.’; Option 3, ‘I would like to kill myself.’; Option 4, ‘I would kill myself if I had the chance.’. In line with the current definition of S^+^/S^−^ in the clinical dataset, we identified the S^+^ group as choosing Option 2, 3, or 4, while participants selecting Option 1 were categorized as the S^−^ group. Therefore, there were 129 participants in S^+^ and 618 participants in S^−^. We did not find a significant group difference in sex and age (ps > 0.075). To make it comparable, we fit the winning choice and mood models from the clinical study.

### Predictive model of suicidal risks

#### Internal validation

To evaluate the out-of-sample predictive utility of computational parameters for STB, we used lasso regression within a repeated nested fivefold cross-validation framework. In each of 100 iterations, the full sample was randomly divided into five folds. For each outer fold, the model was trained on four folds and tested on the remaining fold. Predictor variables were *z*-scored within the training data, and the corresponding training-set means and standard deviations were then applied to normalize the test data. Within each training set, the lasso penalty parameter was selected via an inner 5-fold cross-validation procedure using the minimum mean squared error criterion. The resulting coefficients were then used to generate predictions for the held-out fold. After all outer folds had been completed, the cross-validated predictions for all participants were combined, and model performance was quantified as the Spearman correlation between predicted and observed STB scores, given that suicidal symptom scores (BSI-C) were not normally distributed (Kolmogorov–Smirnov test, p < 0.001). This entire procedure was repeated 100 times to obtain a stable estimate of predictive performance.

#### External validation

To further assess robustness and generalizability beyond the original sample, we conducted an external validation analysis using an independent dataset (*n* = 747). Specifically, regression coefficients and intercepts were averaged across folds and repetitions from the internal validation procedure to derive a stable final model. This model was then applied to the external dataset, using the same predictors (*β*_gain_ and *β*_CR_), to generate predicted scores. External validity was assessed by calculating the Spearman correlation between model-predicted scores and scores on item 9 of BDI.

### Statistical analysis

We performed chi-square, independent-sample *t*-test, or repeated measures ANOVA to test group-related differences. Spearman correlations were used to check correlations among suicidal-related questionnaires, choice data, and mood data. Generalized linear models were conducted for control analysis using MATLAB R2015a. Mediation analysis was conducted using R (4.1.0) and the R package ‘mediation’. All reported tests are two-tailed unless otherwise specified. For the replication of previous findings in the validation dataset, we used one-tailed tests in line with our clinically motivated directional hypothesis. We set the significance level at p = 0.05. Multiple comparisons were corrected using Benjamini–Hochberg false discovery rate (FDR) correction (see Appendix 8 for details).

## Results

### Demographic and clinical characteristics

Overall, sex and age were comparable among S^+^, S^−^, and HC groups (ps >0.157), though S^+^ was significantly younger than S^−^ (*t* = 1.997, p = 0.049). As expected, S^+^ scored significantly higher than S^−^ and HC in suicidal-related scales (e.g., BSI-C; ps <0.001), further validating our grouping of participants. There was no significant difference between S^+^ and S^−^ in illness duration, family history, diagnosis, and various medications use (ps > 0.07), except for other anxiolytics (*χ*^2^ = 5.434, p = 0.020). See Table 1, Appendix 1—table 2 for details.

### Sanity checks

**Figure 2.**
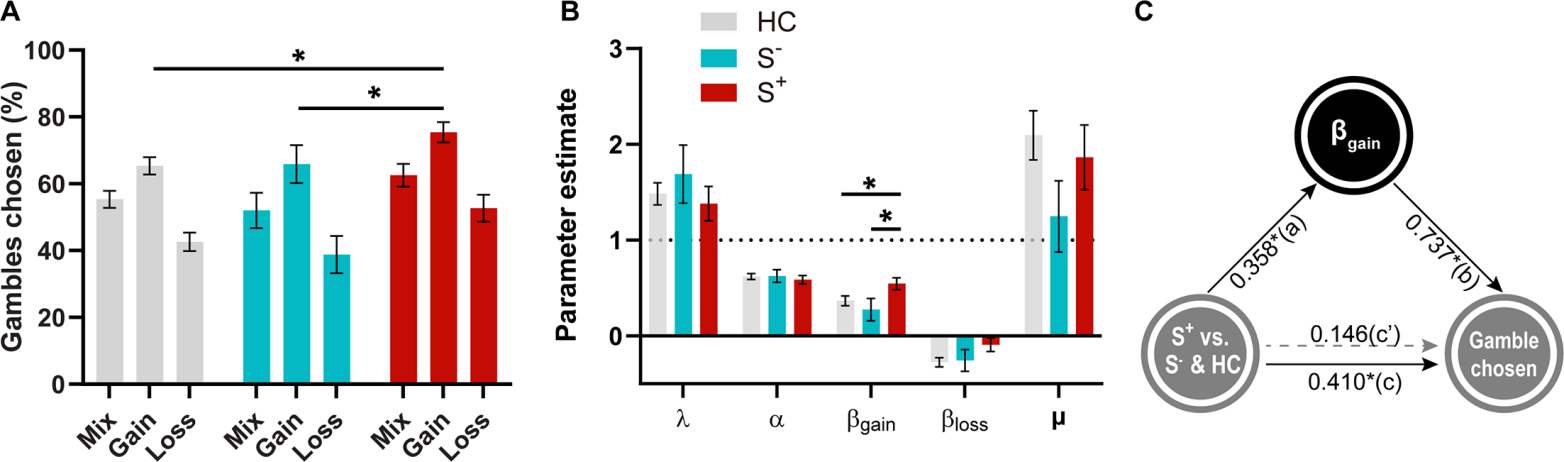
Choice results. (**A**) Group differences in gambling behavior. The gray dots represent the winning model prediction. (**B**) The estimated parameters from the winning choice model differed across groups. S^+^ exhibited stronger approach motivation than S^−^ and HC. (**C**) The mediation model among the group, $\beta _{gain}$, and gambling behavior in the gain condition. The approach parameter mediated the effects of STB group on increased gambling behavior in the gain condition. Abbreviations: HC, healthy control; S^−^, patients without suicidal thoughts and behavior; S^+^, patients with suicidal thoughts and behavior; *p < 0.05.

To ensure engagement and task validation, we performed sanity checks. As expected, we found significant group differences in psychological measurements (ps <0.001), including childhood trauma, emotion regulation, and anxiety/depression (Table 1, Appendix 1—table 2). In addition, we replicated the classic mood-related effects (Blain and Rutledge, 2020; Jangraw et al., 2023): (1) subjects were happier after winning than losing (*t* = 11.001, p < 0.001; Figure 1B) and (2) mood drifted over time (*t* = –3.254, p = 0.001; Figure 1C). As grouping checks, we found a hierarchical pattern of mood level both before the task and across the task (S^+^ < S^−^< HC; for initial mood, *F* = 53.415, p < 0.001; S^+^ vs. S^−^: *t* = –4.525, p < 0.001; S^+^ vs. HC: *t* = –10.427, p < 0.001; S^−^ vs. HC: *t* = –2.634, p = 0.009; Figure 1D; for mean mood, *F* = 28.018, p < 0.001; S^+^ vs. S^−^: *t* = –3.773, p < 0.001; S^+^ vs. HC: *t* = –7.292, p < 0.001; S^−^ vs. HC: *t* = –1.458, p = 0.147; Figure 1E). No significant group difference in mood variation was found (*F* = 1.270, p = 0.284; Figure 1F), which suggests that any parameter difference between groups is unlikely to be explained by mood variance. Moreover, there was no group difference in terms of mood drift effect, or earnings (Figure 1G; ps > 0.276).

### Patients with suicidal thought and behavior approached gambles more than patient controls and healthy controls, while risk attitude was comparable across groups

To replicate previous findings of increased risk behavior in suicidal populations, we conducted a two-way ANOVA on gambling rate with group (S^+^/S^−^/HC) as a between-subject factor and trial type (mix/gain/loss) as a within-subject factor. We found a significant main effect of group (*F* = 3.655, p = 0.028, partial $\eta ^{2}$ = 0.036; Figure 2A), with more gambling behavior for S^+^ than S^−^ (two-sample *t*-test, *t* = 2.145, p = 0.035) and HC (*t* = 2.465, p = 0.015) and comparable gambling behavior between S^−^ and HC (*t* = –0.439, p = 0.661) across the task. We also observed the main effect of trial type (*F* = 51.225, p < 0.001, partial $\eta ^{2}$ = 0.206; gain > mix > loss). We did not observe any significant interaction effect between group and trial type (*F* = 0.270, partial $\eta ^{2}$ = 0.003). Within patients, this group effect on gambling rate remained significant after controlling for sex, illness duration, family history, diagnosis, and various medications use (ps < 0.05), as well as general symptoms (e.g., depression and anxiety; p = 0.024; also see Appendix 1—table 4 and Appendix 1—table 10). Given high correlations among anxiety and depression questionnaires (*r*s > 0.753, ps <0.001), we performed principal components analysis (PCA) to extract main components, where each component explained 86.95%, 7.09%, 3.27%, and 2.68% variance, respectively. To further control for anxiety and depression, linear regression using these components as covariates revealed that the group effect on gambling rate remained significant (p = 0.024; Appendix 1—table 11). There was also no significant age/other anxiolytics use difference in gambling behavior (ps > 0.109; Appendix 1—figure 2).

We next performed a model comparison to select the model that best explains choice data. This analysis revealed that the winning model to formally quantify mechanisms for observed risky behavior is the approach-avoidance prospect theory model (cM3; mean *R*^2^ = 0.37; Table 2). Parameter and model recovery analyses showed that each model and parameter can be identified (see Appendix 5; Appendix 1—figures 5 and 6). As predicted, we found a (marginally) significant group effect in approach parameter (*F* = 2.989, p = 0.053; Figure 2C), with a significantly stronger approach motivation for S^+^ than S^−^ (*t* = 2.217, p = 0.029) and HC (*t* = 2.091, p = 0.038), and comparable between S^−^ and HC (*t* = –0.737, p = 0.463). No other significant group difference in these parameters was found (ps > 0.135). Within patients, this group effect on the approach parameter remained significant after controlling for sex, illness duration, family history, diagnosis, and various medications use (ps < 0.05), as well as general symptoms (e.g., depression and anxiety; p = 0.027; also see Appendix 1—figure 4, Appendix 1—table 10). Linear regression using PCA components as covariates revealed that the group effect on approach parameter remained significant (p = 0.027; Appendix 1—table 11). There was also no significant age/other anxiolytics use difference in gambling behavior (ps > 0.223; Appendix 1—figure 2). Given significant correlations between group, approach parameter, and gambling rate for gain trials (ps < 0.017), we further conducted a mediation analysis with the assumption of the mediating effect of approach motivation of suicidality on the risk behavior. Given that we aimed to test the effect of STB, with S^−^ and HC as controls, and S^−^ and given that HC did not differ in gambling behavior or in the approach parameter, we merged these two groups for the mediation analysis. Results supported our hypothesis (*a* × *b* = 0.321, 95% CI = [0.070, 0.549], p = 0.016; Figure 2C), confirming that suicidal thoughts and behavior increase risk behavior through stronger approach motivation.

**Table 2.** Choice model comparison.

Model #	Model specification	# of parameters	Δ BIC	Mean *R*^2^	Δ BIC for each group		
					HC	S^−^	S^+^
1	*µ*	1	3873.16	0.08	2272.48	370.19	1230.49
2	*λ*, *α*, *µ*	3	3153.79	0.18	1822.07	263.97	1067.75
3	***λ*, *α*, *β*_gain_, *β*_loss_, *µ***	5	0	0.37	**0**	**0**	**0**

^Δ^BIC, Bayesian information criterion relative to the winning model (cM3); HC, healthy control; S^−^, patients without suicidal thoughts and behavior; S^+^, patients with suicidal thoughts and behavior.

### Mood sensitivity to certain rewards was reduced in patients with suicidal thought and behavior compared to patient controls and healthy controls

**Figure 3.**
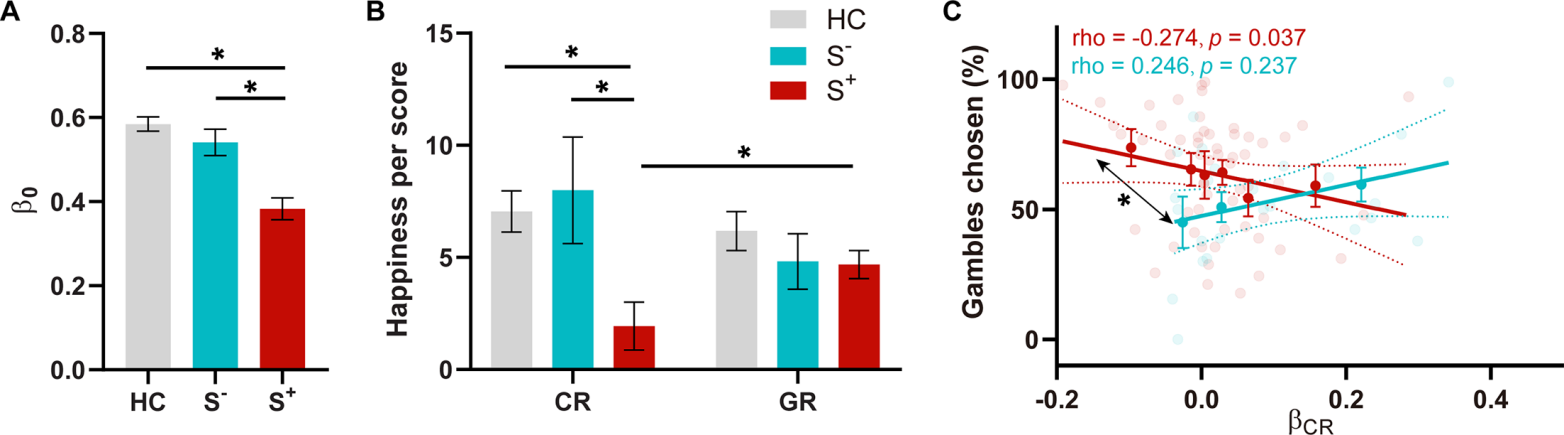
Effect of Suicidal thoughts and behavior on mood dynamics. (**A**) Group difference in mood baseline, *β*_0_. (**B**) Group differences in mood sensitivity to certain reward (CR) and gamble reward (GR). (**C**) Correlational difference in S^−^ and S^+^ between mood sensitivity to CR and gambling behavior. The lighter, semi-transparent dots represent individual participants, while the dark dot with an error bar indicates the mean of binned scores (for illustration purposes only). Abbreviations: CR, certain reward; GR, gamble reward; HC, healthy control; S^−^, patients without suicidal thoughts and behavior; S^+^, patients with suicidal thoughts and behavior; *p < 0.05.

Next, we turned to mood model comparison. We observed inconsistent mood winning models for different groups (Table 3), suggesting an effect of STB on mood dynamics. Given that the focus of the current study was STB effect, especially for the S^+^ group, with the baseline control of S^−^ and HC groups, we specially focused on the winning model from the S^+^ group. Parameter and model recovery analyses showed that each model and parameter can be identified (see Appendix 5; Appendix 1—figures 5 and 6 and Appendix 1—table 7). The winning mood model from S^+^ assumed that momentary mood fluctuations were explained by the recency-weighted average of certain reward (CR) and the gamble reward (GR; mM3; mean *R*^2^ = 0.42; Table 3). Overall, both CR and GR weights were significantly higher than 0 (CR: *t* = 8.033, p < 0.001; GR: *t* = 9.853, p < 0.001). The baseline parameter *β*_0_ was significantly correlated with the initial mood (rho = 0.580, p < 0.001), validating this model. We also replicated previous depression-related findings (Rutledge et al., 2017): depression symptom measured by Beck Depression Inventory (BDI) was negatively correlated with the baseline mood parameter $\beta _{0}$ (rho = –0.530, p < 0.001; Appendix 1—figure 7). We found significantly lower *β*_0_ in S^+^ than S^−^ (*F* = 22.861, p < 0.001; *t* = –3.513, p < 0.001) and HC (*t* = –6.606, p < 0.001), which mirrors the lower initial mood pattern. Importantly, a two-way ANOVA on mood parameters with group (S^+^/S^−^/HC) as a between-subject factor, event type (CR/GR) as a within-subject factor showed a significant main effect of group (*F* = 3.835, p = 0.023, partial $\eta ^{2}$ = 0.037), with lower mood sensitivity for S^+^ than S^−^ (*t* = –2.080, p = 0.041) and HC (*t* = –2.758, p = 0.006) and comparable between S^−^ and HC (*t* = –0.110, p = 0.913). We also observed a significant interaction effect between group and event type (*F* = 4.283, p = 0.015, partial $\eta ^{2}$ = 0.041; Figure 3B). Simple effect analysis revealed that S^+^ group exhibited significantly lower mood sensitivity to CR as compared to GR (*F* = 4.823, p = 0.029, partial $\eta ^{2}$ = 0.024), while there was no significant CR–GR difference in S^−^ (although trendy; *F* = 2.783, p = 0.097, partial $\eta ^{2}$ = 0.014) and HC (*F* = 0.989, p = 0.321, partial $\eta ^{2}$ = 0.005). This interaction was driven by the group difference in CR (*F* = 6.085, p = 0.003, partial $\eta ^{2}$ = 0.058) rather than in GR (*F* = 0.801, p = 0.450, partial $\eta ^{2}$ = 0.008). Specifically, S^+^ showed lower mood sensitivity to CR than S^−^ (*t* = –2.661, p = 0.009) and HC (*t* = –3.381, p < 0.001), while S^-^ and HC were comparable (*t* = 0.450, p = 0.679), suggesting S^+^ was specifically more insensitive to certain outcomes than gamble outcomes. No significant main event type (CR vs. GR) effect was found (*F* = 0.285, p = 0.594, partial $\eta ^{2}$ = 0.001). Within patients, this group effect on *β*_CR_ remained significant after controlling for gambling rate, earnings, mood-related outcome effect, mood drift effect, sex, illness duration, family history, diagnosis, and various medications use (ps < 0.032), as well as general symptoms (e.g., depression and anxiety; p = 0.001; also see Appendix 1—figure 4 and Appendix 1—table 10). Linear regression using PCA components as covariates revealed that the group effect on this mood parameter remained significant (p = 0.001; Appendix 1—table 11). There was also no significant age/other anxiolytics use difference in gambling behavior (ps > 0.582; Appendix 1—figure 2). These results indicate decreased mood sensitivity for certain rewards in suicidal populations.

**Table 3.** Mood model comparison.

Model #	Model specification	# of parameters	Δ BIC	Mean *R*^2^	Δ BIC for each group		
					HC	S^−^	S^+^
1	*β*_0_, *β*_CR_, *β*_EV_, *β*_RPE_, *γ*	5	–106.77	0.48	–182.04	32.54	42.73
2	*β*_0_, *β*_CR_, *β*_EV_, *β*_RPE_, *γ*_CR_, *γ*_EV_, *γ*_RPE_	7	140.00	0.54	–69.40	83.20	126.20
3	***β*_0_, *β*_CR_, *β*_GR_, *γ***	4	0	0.42	0	0	**0**
4	*β*_0_, *β*_CR_, *β*_GR_, *γ*_CR_, *γ*_GR_	5	–146.81	0.48	–272.15	26.37	98.97
5	*β*_0_, *β*_CR_, *γ*	3	2395.62	0.18	1379.96	264.87	749.79
6	*β*_0_, *β*_GR_, *γ*	3	403.46	0.34	228.69	21.24	153.52

^Δ^BIC, Bayesian information criterion relative to the winning model in S^+^ group (mM3); HC, healthy control; S^−^, patients without suicidal thoughts and behavior; S^+^, patients with suicidal thoughts and behavior.

In addition to the winning model (mM3) from S^+^ group, we also checked results from the classic mood model (mM1). Overall, we replicated previous findings (Appendix 1—figure 8): (1) mood sensitivity to CR, EV, and RPE were all significantly higher than 0 (ps < 0.001); (2) higher weight for RPE than EV (*t* = 5.760, p < 0.001). Although no significant group difference between S^+^ and S^−^ was found in each parameter (ps > 0.115), we replicated significant correlation between BSI-C and *β*_CR_ (rho = –0.239, p = 0.030). To explore why the classic mood model (mM1) did not outperform the CR–GR model, we examined expectation effect on mood, as previous literature showed impaired value expectation in patients with STB (Dombrovski et al., 2013). Our data suggest a lower mood sensitivity to RPE relative to EV in S^+^ than HC (Appendix 1—figure 9; significant interaction between group and EV/RPE: *F* = 3.422, p = 0.035; with stronger mood sensitivity to RPE than EV in HC (*F* = 36.658, p < 0.001), while no such significant difference in S^+^ (*F* = 1.161, p = 0.283) and S^−^ (*F* = 3.009, p = 0.084)). Equal weights on EV and RPE suggest that expectations cancel out as RPE is the difference between the outcome and EV, resulting in outcome only. Then, we additionally fit a mood model with CR, GR, and EV components (Appendix 1—figure 9). We expect less negative mood sensitivity to EV in S^+^ than HC. As expected, in addition to replication of our main results (ps <0.045; *R*^2^ for this model: 0.487), we observed a less negative mood sensitivity to EV in S^+^ than HC (*t* = 2.302, p = 0.023), which explains why the winning model shifts to mM3. Given that mM7 (splitting GR into better and worse terms) performed better than our winning model (mM3) in the S^−^ and HC groups, we also checked results from this model (Appendix 1—figure 10 and Appendix 1—table 6). Again, we found that S^+^ had significantly lower *β*_CR_ than S^−^ and HC (for group effect: *F* = 44.660, p = 0.011; S^+^ vs. S^−^: *t* = –2.659, p = 0.009; S^+^ vs. HC: *t* = –2.589, p = 0.010; S^−^ vs. HC: *t* = 1.059, p = 0.292) and significant correlation between BSI-C and *β*_CR_, (rho = –0.297, p = 0.006) among patients, suggesting the robustness of mood sensitivity to certain reward in suicidal people. The marginally significant group effect in approach parameter (p = 0.053) remains marginally significant after correction (p = 0.068). In addition to this, all results of interest, including gambling chosen, approach parameter, and mood sensitivity to CR, remained significant with FDR correction (ps ≤ 0.05; Appendix 8).

### Suicidal thought and behavior effect on gambling was mediated by mood sensitivity to certain rewards

To examine the association between risk behavior and atypical mood dynamics in suicidal patients, we then tested the correlation between participants’ gambling rate and mood sensitivity to certain reward (*β*_CR_) in S^+^. We found significant negative correlation between gambling rate and *β*_CR_ in S^+^ (rho = –0.274, p = 0.037; Figure 3C), suggesting the lower mood sensitivity to certain reward, the more gambling behavior suicidal patients made. We did not observe such a significant correlation in S^-^ (rho = 0.246, p = 0.237) and there was significant correlational difference between S^+^ and S^−^ (*Z* = –2.109, p = 0.017; Millner et al., 2019), suggesting the suicidal-specific association of mood and choice.

### Replication of suicidal-related results in an independent dataset (*n* = 747)

**Figure 4.**
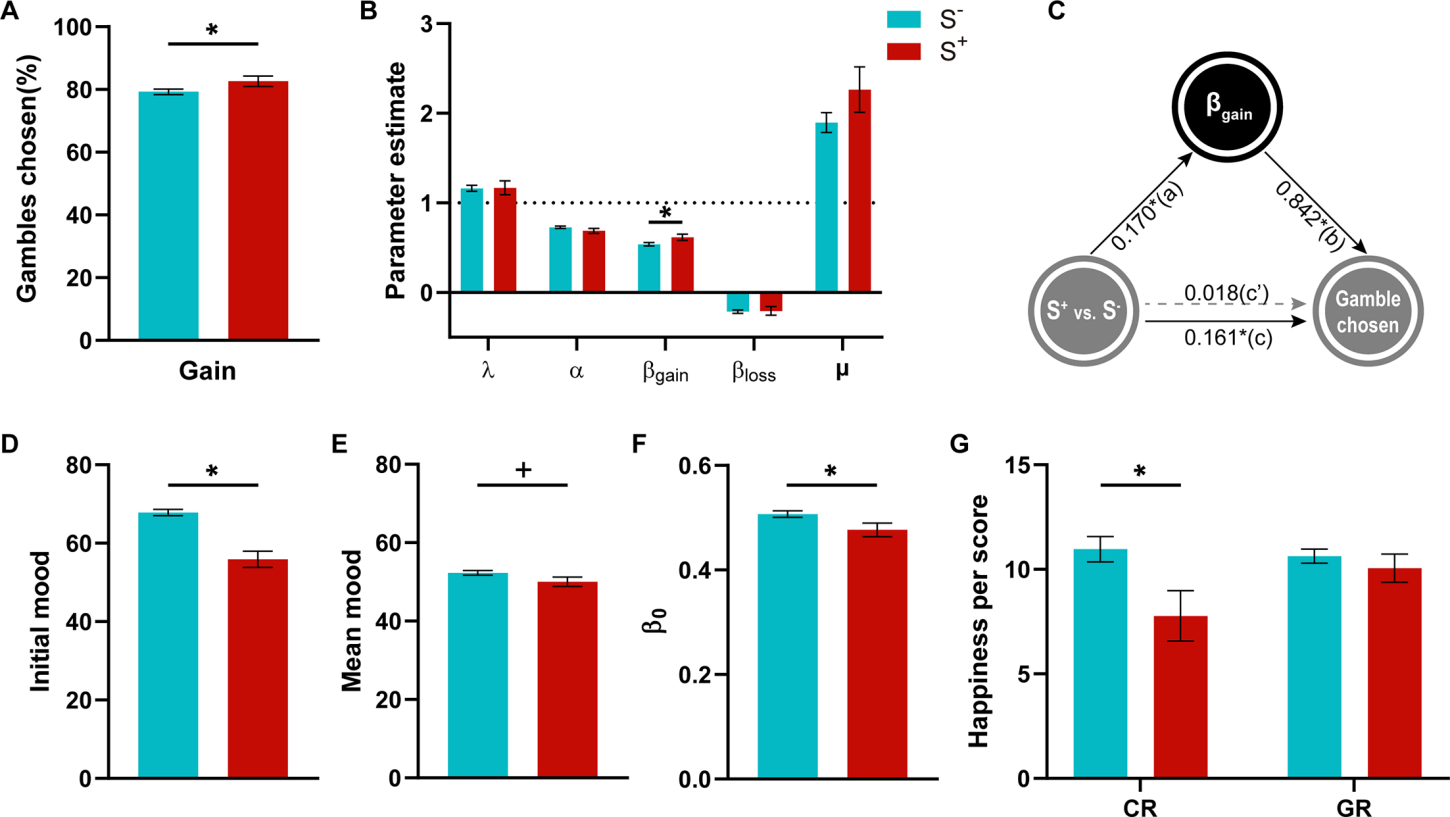
Validation of suicidal-related results in an independent dataset of general populations (*n* = 747). (**A**) Group difference in gambling behavior in the gain domain. (**B**) The estimated parameters from the winning choice model (pseudo *R*^2^ = 0.479) differed across groups, with higher approach behavior for S^+^. (**C**) The mediation model among the group, $\beta _{gain}$, and gambling behavior in the gain condition. The approach parameter mediated the group effect on increased gambling behavior in the gain condition. (**D**) Group difference in mood before the task shows weakened mood in S^+^. (**E**) Group difference in average mood displays lower mood experience in S^+^. (**F, G**) The estimated parameters from the CR–GR mood model (mean *R*^2^ = 0.588). (**F**) Group difference in mood baseline, *β*_0_. (**G**) Group differences in mood sensitivity to certain reward (CR) and gamble reward (GR). Abbreviations: S^−^, general participants without suicidal thoughts and behavior; S^+^, general participants with suicidal thoughts and behavior; *p < 0.05, ^+^p < 0.1.

Next, we collected online data on general volunteers to replicate our findings. In this large online dataset, we found lower mood experience in general volunteers who replied non-negatively to the Suicidal item of the BDI (S^+^). Regarding the initial mood rating (before the task), S^+^ exhibited significantly lower mood than S^−^ (*t* = –6.077, p < 0.001; Figure 4D). There was a trend for lower mood experience across time in S^+^ than S^−^ (*t* = –1.600, p = 0.055; Figure 4E). Critically, we identified a significantly increased gambling behavior in S^+^ than S^−^, especially in the gain domain (*t* = 1.668, p = 0.048; Figure 4F). Approach-avoidance prospect theory model (mean pseudo*R*^2^ = 0.479) revealed a significantly heightened approach parameter in S^+^ than S^−^ (*t* = 1.762, p = 0.039; Figure 4B), but not any other choice parameters (ps > 0.172). We also replicated the previous mediation result that STB increased risk behavior through stronger approach motivation (*a* × *b* = 0.143, 95% CI = [0.016, 0.288], p = 0.031; Figure 4C). Regarding the CR–GR mood model (mean *R*^2^ = 0.588), we observed significantly lower *β*_0_ in S^+^ than S^−^ (*t*=–2.018, p = 0.022; Figure 4F). Mood sensitivity to CR (*t* = –2.237, p = 0.013; Figure 4G), but not GR (*t* = –0.187, p = 0.473; Figure 4G), was significantly reduced in S^+^ than S^−^. After controlling for depression severity using our established bifactor model (see ref 60 for details), these results remained significant (ps ≤0.050), except for a marginally significant effect of group on gambling behavior (p = 0.059). Despite a trend, this effect with covariates of depression-related questionnaires is strong in our clinical cohort (p = 0.024). This suggests that the link between suicidality and risky behavior persists above and beyond general depressive symptoms.

These validation results suggest that our computational markers can generalize to the general population. However, we did not observe any significant correlation between mood sensitivity to CR and gambling behavior (ps > 0.389), which suggests that the link between mood sensitivity to CR and gambling behavior may be specifically observable in suicidal patients. Alternatively, this non-replicated result may also reflect sample-specific or unstable effects, which need to be interpreted with caution.

### Computational parameters were predictive of suicidal risks

To examine whether task-derived computational measures carry predictive information related to suicidal ideation (BSI-C) beyond single-parameter associations, we performed an additional multivariate prediction analysis using lasso regression within a cross-validation framework. Across 100 repetitions of fivefold cross-validation, STB was significantly predicted by the computational parameters (mean $r$ = 0.205, all ps < 0.039; Table 4), including approach motivation and mood sensitivity to certain rewards, across all participants, including both patients and healthy controls. Importantly, this predictive model generalized to the online sample (*n* = 747), where model-predicted scores were significantly correlated with scores on item 9 of BDI (*r* = 0.073, p = 0.045). We further confirmed the robustness of this predictive effect using 10-fold cross-validation, which yielded a similar pattern of results (Table 4). By contrast, predictive models based on choice-only or mood-only parameters did not show reliable generalization to the external validation sample (ps > 0.191), suggesting that the predictive signal emerged from the joint contribution of choice- and mood-related computational measures rather than either domain alone. In sum, these internal and external validation analyses corroborated the robustness of the predictive model of suicidal risks using computational markers.

**Table 4.** Suicidal risk prediction from computational parameters.

Internal validation (*n* = 201)				External validation (*n* = 747)	
Cross-validation	Rho (mean ± SD)	Rho [min, max]	p values	Rho	p value
Fivefold	0.205 ± 0.016	[0.146, 0.230]	<0.039	0.073	0.045
Tenfold	0.207 ± 0.011	[0.172, 0.226]	<0.014	0.073	0.045

## Discussion

The current study tested cognitive and affective computational mechanisms for increased risk behavior in adolescent patients with suicidal thoughts and behaviors (STB), with a control group including adolescent patients without STB and sex/age-matched healthy control (HC). First, we observed an increased gambling behavior and a lower overall mood in STB patients (S^+^), as compared to non-STB patients (S^−^) and HC, replicating previous findings (Sastre-Buades et al., 2021; Perrain et al., 2021; Richard-Devantoy et al., 2014; Ortin et al., 2012). Second, using an approach-avoidance prospect theory model, we found heightened approach motivation in S^+^ than S^−^ and HC, which explained increased gambling choices for STB, suggesting an over-reactivity of the approach system to approach risky options. Third, using a momentary mood model, we showed that lower mood sensitivity to certain outcomes in S^+^ compared to S^−^ and HC, which was driven by lower mood sensitivity to certain outcomes in S^+^ than S^−^ and HC. These computational markers generalized to the general population (*n* = 747). Importantly, mood hyposensitivity to certain reward specifically correlated to more gambling behavior in S^+^, offering a mood computational account for increased risk behavior in STB. Beyond these specific findings, this work highlights the broader utility of combining computational modeling with momentary mood measures to better characterize behavioral differences relevant to psychiatric symptoms, and to show how choice and mood data can jointly inform our understanding of psychiatric phenomena.

Our results suggest a unique reason for the twofold observations that STB patients display an increase in both risk taking and impulsivity, defined as a tendency to act quickly without planning while failing to inhibit a behavior that is likely to result in negative consequences (Horesh et al., 1999; Javdani et al., 2011; Kingsbury et al., 1999; Renaud et al., 2008; Dayan et al., 2006). Indeed, we did not observe a difference in risk attitude (e.g., risk aversion and loss aversion) per se between STB and controls but instead a higher approach behavior toward largest rewards (i.e., the lotteries) in STB patients. This would result from the value-independent term in the model that represents forms of approach in the face of gains (Rutledge et al., 2015; Bushong et al., 2010; Guitart-Masip et al., 2012). Such approach actions are elicited without regard to their actual contingent benefits and therefore correspond to impulsive behavior. A substantial body of research has shown that impulsivity, as assessed either through questionnaires or clinical observations, is a key predictor for STB (for a review, see Franklin et al., 2017). Our study employed computational modeling to quantitatively elucidate the altered approach-system processing for increased risky behavior in STB, offering enhanced predictive power and generalizability (Huys et al., 2016). On the other hand, contrary to the proposal of atypical avoidance system (Dombrovski and Hallquist, 2022; Dombrovski and Hallquist, 2017), we did not observe significant group difference in avoidance, which may be attributed to the different involvement of the motivational system in learning and non-learning contexts (Rutledge et al., 2016b; Allen et al., 2019). In our model specification, motivational systems work in a value-independent way in the non-learning context. Consistent with the view that suicide is an escape from intolerable affective states, (O’Connor and Nock, 2014), risky behavior in suicidal individuals may be rewarding. In clinical practices, understanding the distortion of the approach system in STB may encourage mental health professionals to closely monitor patients who exhibit heightened approach tendencies. Such vigilance may enable early detection of risk-related behaviors, thus facilitating timely intervention strategies tailored to mitigate impulsivity-driven actions that may elevate the likelihood of STB.

Consistent with suicidal-related theories (O’Connor and Nock, 2014; Stewart et al., 2019a; Hall et al., 2024) and as summarized by Millner et al., 2020, we observed lower mood levels in patients with STB, regarding both initial happiness and mood baseline (the latter corresponding to the steady state mood converges to). More importantly, STB patients’ mood was less sensitive to certain outcomes than control without STB, which would lead them to take more risk regardless of the gain at stake and therefore to potentially experience more suboptimal outcomes than controls (Sastre-Buades et al., 2021). Although no direct causal link was established between STB and happiness ratings in response to wins or losses, recent literature has documented associations between STB and anhedonia symptoms (albeit with mixed evidence; for a review, see Hall et al., 2024), where anhedonia can be assessed through affective reactivity to wins vs. losses (Vanhasbroeck et al., 2021). Our findings thus provide support for the presence of anhedonia in STB, particularly in response to certain outcomes. Surprisingly, mood model-based analysis did not support the effect of expectations and prediction errors on mood in healthy people (the ‘CR-EV-RPE model’; Rutledge et al., 2015; Rutledge et al., 2014; Wagner et al., 2021), but suggest instead a dissociation between certain outcomes and lottery outcomes (the ‘CR–GR model’). These two models differed with respect to the inclusion of reward expectation terms, the former including it unlike the latter. This difference can be explained by the lower expected value signal in patients with STB (Dombrovski et al., 2013), resulting in insufficient expectation representations of the gamble option to influence mood dynamics. An alternative explanation could be the duration of the chosen option display which was considerably lower in our design than in other mood studies (e.g., 0.5 s in our study vs. 6 s in Rutledge et al., 2014), which would not leave enough time for expectation to be built. It is also possible that the current winning model was specific to adolescents. Given that Rutledge et al., 2017 supported the ‘CR-EV-RPE model’ in adults with depression, our study with adolescent populations may suggest a developmental change for mood sensitivities. Within the winning CR–GR model, we observed that S^+^ specifically exhibited lower mood sensitivity to CR than GR, which was driven by mood hyposensitivity to CR in S^+^ than S^-^ and HC. This mood insensitivity was associated with STB severity, which was replicated when using the CR-EV-RPE model. Importantly, we found that mood hyposensitivity to certain reward was specifically correlated to gambling behavior in patients with STB, suggesting the potential mood computational mechanism for increased risk behavior in STB. As for clinical practices, CR-based anhedonia linked to CR (computational reactivity) in STB may prompt mental health professionals to closely monitor patients who exhibit mood insensitivity to certain daily events. This proactive monitoring could aid in identifying and addressing risk-related behaviors early on.

With replication in an independent dataset with large sample size (*n* = 747), this study provides robust evidence of the affective and cognitive computational mechanisms underlying heightened risky behavior in adolescents with STB. In addition, these results remained significant after controlling for demographics, social and clinical variables, medication factors, and the timing of suicidal events (Appendices 3 and 4). However, this study could not differentiate between suicidal thoughts and suicidal behaviors. Although it has been shown that they represented different decision-making processes with different neural underpinnings (Schmaal et al., 2020; Saffer and Klonsky, 2018; Jollant et al., 2023), our data did not reveal significant differences between them (see Appendix 2). Future research would benefit from examining these distinctions at the neural level. Nonetheless, by combining the suicidal ideation and suicidal attempt groups into a single STB group (Glenn et al., 2019; Glenn et al., 2017; Millner et al., 2019; Miller et al., 2024; Eisenlohr-Moul et al., 2018; Miller et al., 2017), our findings highlight why adolescents with suicidality exhibit a preference for risky behavior. These findings carry important clinical implications for early prevention of adolescent suicidality. Notably, this study, like many traditional studies on suicidality (Glenn et al., 2019; Glenn et al., 2017; Millner et al., 2019; Tsypes et al., 2024; Kleiman et al., 2017), does not seek to elucidate the affective and cognitive mechanisms underlying fluctuations in suicidal thoughts. Given the inherently variable nature of suicidal ideation, recent research has increasingly adopted ecological momentary assessments to capture real-time variations in suicidal ideations (Miller et al., 2017; Wang et al., 2024; Stewart et al., 2019b). While such methods can help predict when suicidal ideation may arise, they fall short of explaining the underlying mechanisms driving these thoughts. In contrast, our approach, consistent with traditional literature (Glenn et al., 2019; Glenn et al., 2017; Millner et al., 2019; Miller et al., 2024; Eisenlohr-Moul et al., 2018; Tsypes et al., 2024), is directed at understanding why individuals with STB are more inclined toward risky behavior. We acknowledge the interaction between environmental stressors and the occurrence of STB, noting that suicidal severity often diminishes once the stressor is removed (Eisenlohr-Moul et al., 2018; Dombrovski and Hallquist, 2022). This is a crucially important issue in current psychiatric research. For instance, patients with MDD sometimes experience depressive episodes, particularly in response to stressful events. However, collecting data during STB is both impractical and ethically challenging. Our grouping approach assumes trait-driven STB: individuals with a history of STB, despite not during the experiment, represent a cluster of suicidal-related traits (Dombrovski and Hallquist, 2022; Millner et al., 2020). Sensitivity analyses for STB timeframe support this assumption (see Appendix 3). We also recognize that these affective and cognitive impairments may worsen under stress (Eisenlohr-Moul et al., 2018; Dombrovski and Hallquist, 2022). Future studies would benefit from investigating how acute stress influences the propensity for risky behavior in individuals with STB.

Given that STB is a challenging multifactorial phenomenon, the development of a formal theory to quantify suicide seems necessary (Dombrovski and Hallquist, 2022; Karvelis and Diaconescu, 2022; Bredemeier and Miller, 2015). Our cognitive and affective computational insights may pave the way for such a formal theory. Although previous literature has shown various cognitive impairments (Richard-Devantoy et al., 2014), for example, executive function, in STB (Klonsky et al., 2018), our work is the first to quantify mood dynamics impairment and their behavioral consequences, providing insight into potential targets to prevent and intervene in STB. Our results indeed provide a computational mechanism for the main theories of suicide, linking low mood to suicidal behaviors. Suicidal behavior is conceived to result from an intention shaped by various motivational factors (e.g., feeling of entrapment, belongingness, burdensomeness [O’Connor and Kirtley, 2018]). The suicidal intent may then progress to suicidal behavior, which is thought to be moderated by impulsive decisions (e.g., Bredemeier and Miller, 2015). A possibility is that the approach component becomes excessive as the suicidal intent emerges. These findings provide new insights into the putative dynamics underpinning STB and offer potential markers for the early prediction, screening, detection, and prevention of suicidal behavior. These results would explain the observed increase in risk-taking behavior in STB such as substance use, early onset of sexual intercourse, and physical fighting independent of psychiatric diagnosis.

Several limitations are worth mentioning. First, our cross-sectional findings are of correlational nature. Causal relationships remain to be tested in a longitudinal study. Second, although we assumed that increased risky behavior in STB was suboptimal, the current task was not suited to test this, given the task design of random feedback for gambling options. Future work in learning paradigms, where optimality is well defined, may be better suited to test earnings-based links to STB. Third, despite replicating our main results in an independent dataset (*n* = 747), the modest S^−^ subgroup size (*n* = 25) has a limited statistical power. Next, we did not evaluate the noise in our estimate for example, by assessing the test-retest reliability on the task parameters, and it is indeed possible that the parameter estimate is somehow noisy.

To conclude, this study examined cognitive and affective computational mechanisms underlying increased risk behavior in adolescent patients with suicidal thoughts and behaviors. Given very limited predictive abilities of suicide from previous risk-factor investigations (Franklin et al., 2017), our study offers a potential new perspective of mood, at the core of STB, and reveals a relationship between low mood sensitivity to certain reward and an increased risk behavior in STB, possibly suggesting dysfunctional dopaminergic and serotonergic systems. Our findings suggest that computational measures may capture variance related to suicidal tendency in adolescents and thus may be relevant for future work on early identification and prevention of suicidality.

## Figures and Tables

**Appendix 1—figure 1.**
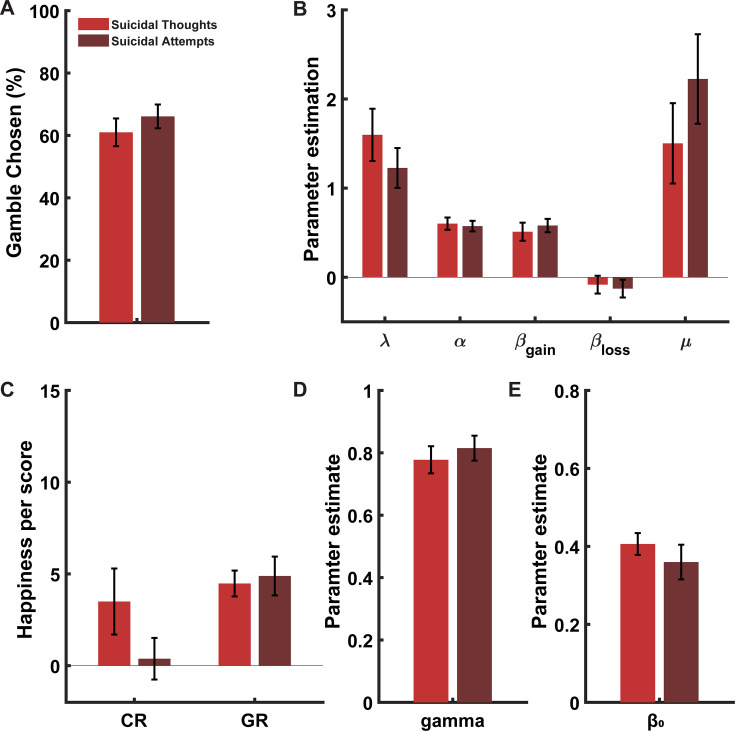
Suicidal attempts vs. suicidal thoughts.

**Appendix 1—figure 2.**
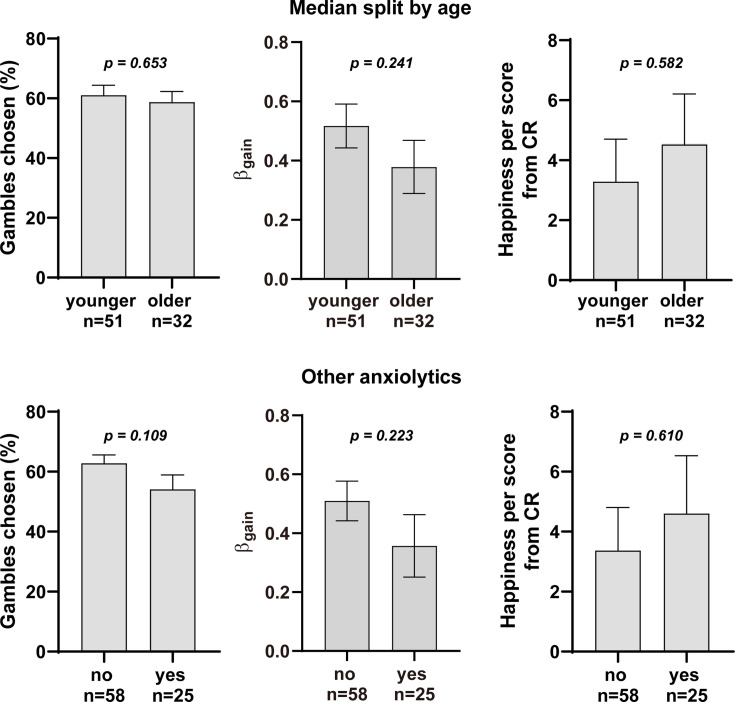
Control analysis for age and other anxiolytics in patients, which were significant between groups.

**Appendix 1—figure 3.**
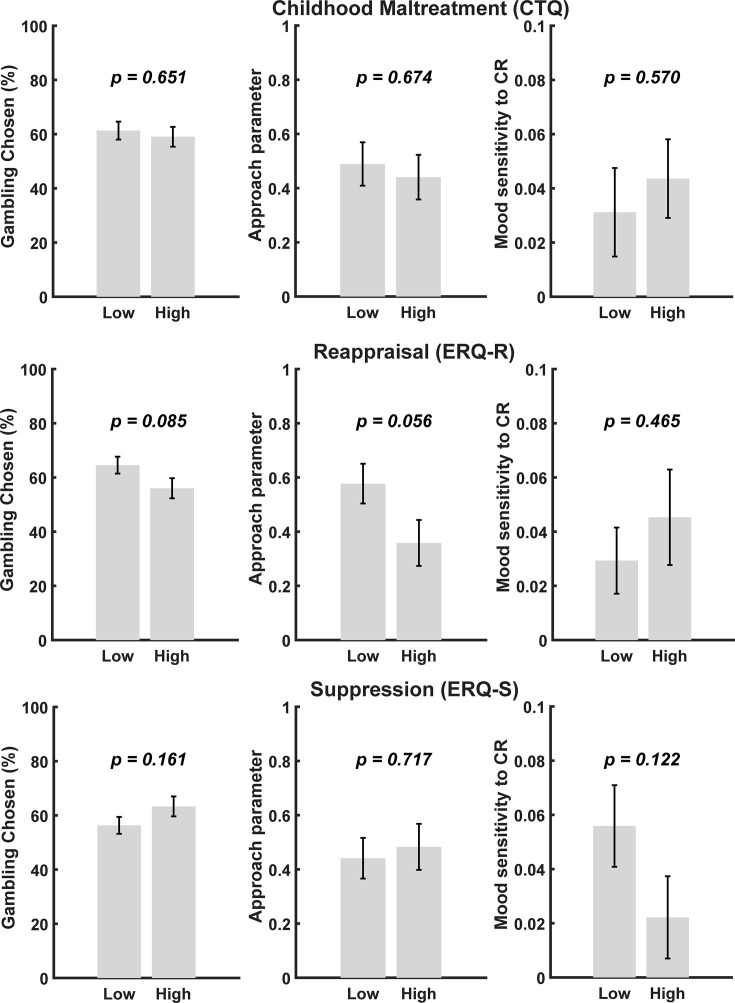
Control analysis for childhood maltreatment and emotion regulation problems in patients, which were significant between groups.

**Appendix 1—figure 4.**
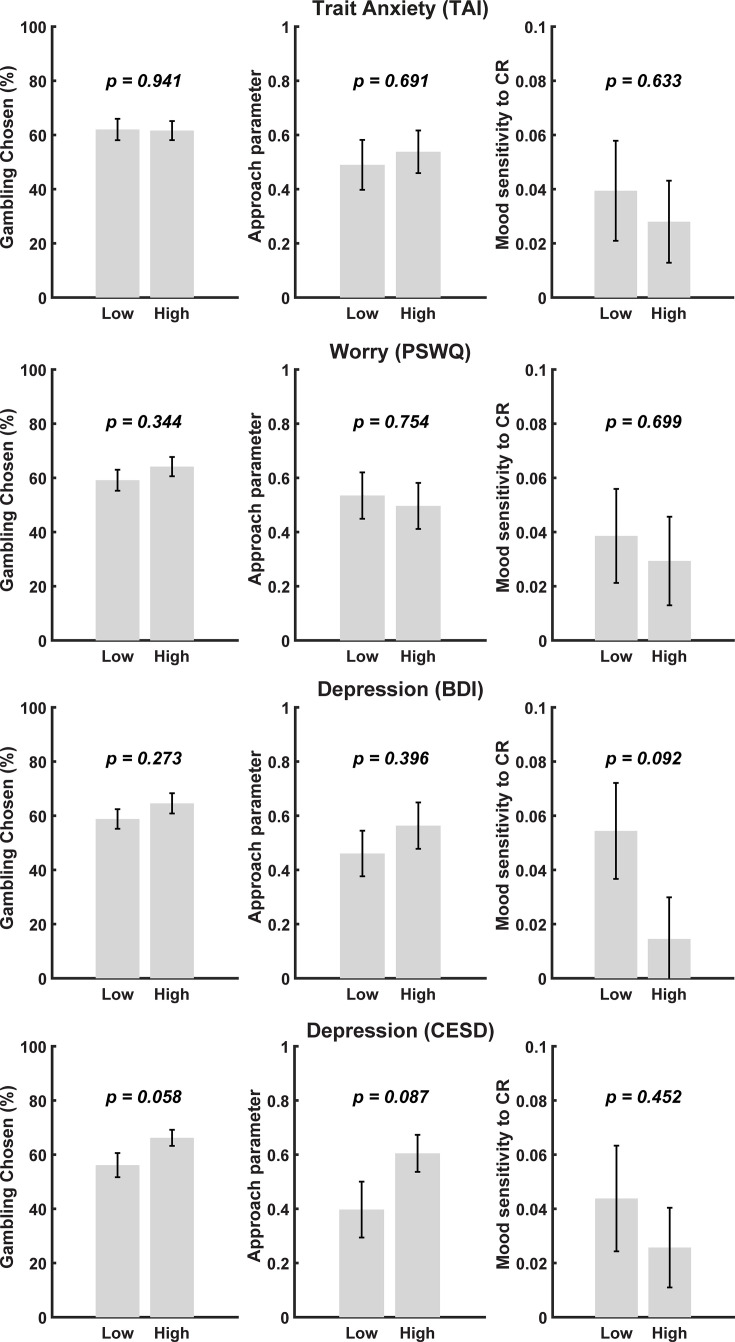
Control analysis for depression/anxiety symptoms in patients, which were significant between groups.

**Appendix 1—figure 5.**
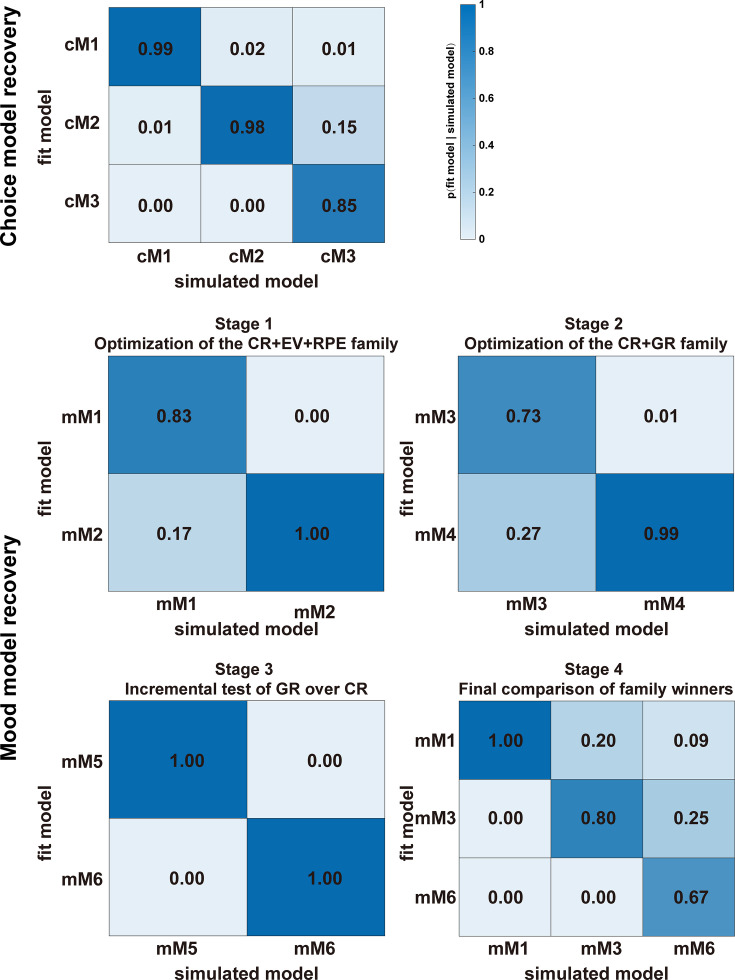
Choice and mood model recovery. Mood model recovery was performed in a stage-by-stage manner, mirroring the model comparison procedure used in the main analyses because each model comparison step addresses a specific question and doing so prevents dispersion of model evidence across multiple similar models. Specifically, rather than entering all mood models into a single recovery space, recovery was evaluated separately within each comparison space used for model selection. Thus, each confusion matrix reflects recovery among only the models that were directly compared at that stage.

**Appendix 1—figure 6.**
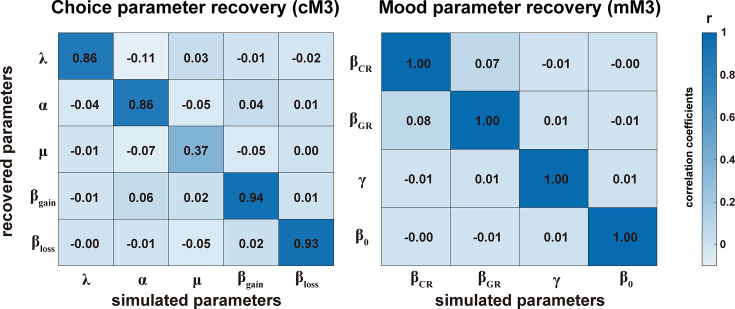
Parameter recovery for the winning choice and mood models (cM3; mM3).

**Appendix 1—figure 7.**
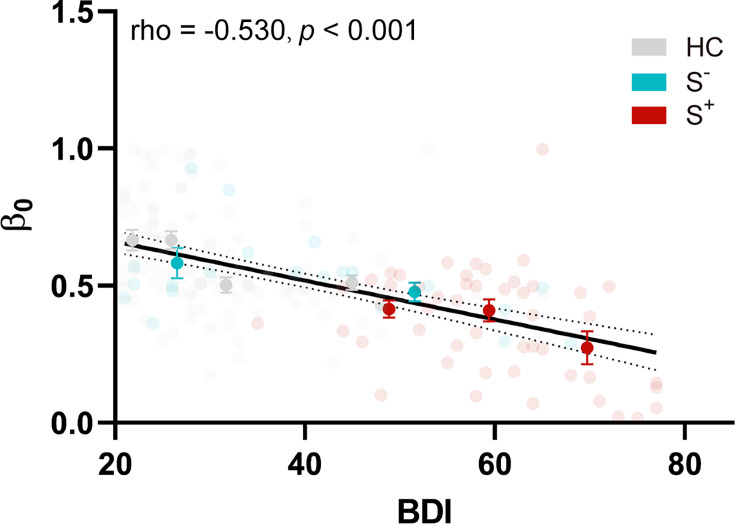
Replication of Rutledge et al., 2017’s findings using BDI. Depression symptom measured by BDI was negatively correlated with the baseline mood parameter.

**Appendix 1—figure 8.**
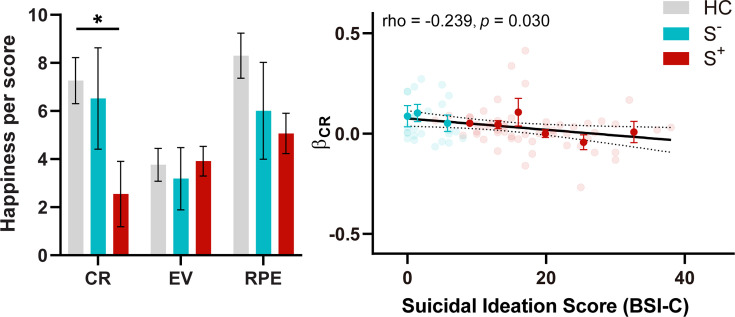
RPE model results (M1). (**A**) Group differences in mood sensitivity to certain reward (CR), expected value (EV), and reward prediction error (RPE). (**B**) Correlation between Suicidal Ideation score at current time (BSI-C) and mood sensitivity to CR. Abbreviations: HC, healthy control; S^−^, patients without suicidal thoughts and behavior; S^+^, patients with suicidal thoughts and behavior; BSI-C, Beck Scale for Suicidal Ideation at the current time; *p < 0.05.

**Appendix 1—figure 9.**
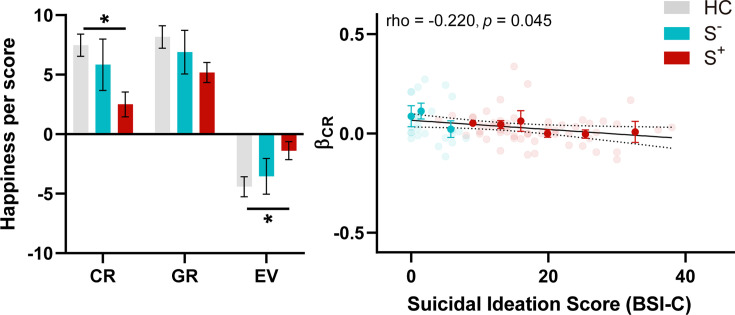
Expectation effect on mood. (**A**) Group differences in mood sensitivity to certain reward (CR), gamble reward (GR), and expected value (EV). (**B**) Correlation between Suicidal Ideation score at current time (BSI-C) and mood sensitivity to CR. Abbreviations: HC, healthy control; S^−^, patients without suicidal thoughts and behavior; S^+^, patients with suicidal thoughts and behavior; BSI-C, Beck Scale for Suicidal Ideation at the current time; *p < 0.05.

**Appendix 1—figure 10.**
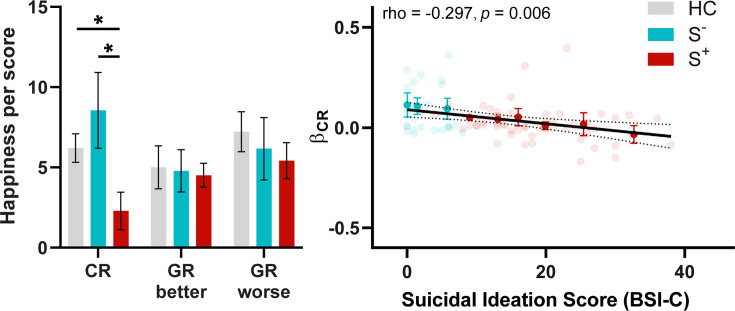
Results from M5. (**A**) Group differences in mood sensitivity to certain reward (CR), better gamble reward (GRbetter), and worse gamble reward (GRworse). (**B**) Correlation between Suicidal Ideation score at current time (BSI-C) and mood sensitivity to CR. Abbreviations: HC, healthy control; S^−^, patients without suicidal thoughts and behavior; S^+^, patients with suicidal thoughts and behavior; BSI-C, Beck Scale for Suicidal Ideation at the current time; *p < 0.05.

**Appendix 1—figure 11.**
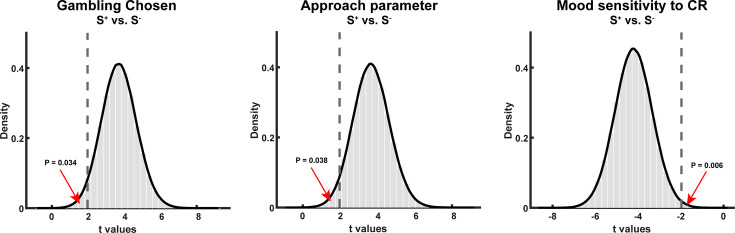
Permutation tests. We conducted permutation tests (1,000,000 iterations) to evaluate the robustness of our main results, ensuring consistency with the same normal distribution, and sample size. Specifically, in each permutation, we randomly drew five samples from the S^+^ group and repeated this process 20 times to construct a suicidal group (100 samples). The same procedure was applied to the S^−^ group to construct a control group. We then calculated the *t*-values between the two constructed groups on variables of interest, including the proportion of gambling choices, the approach parameter, and mood sensitivity to certain rewards (CR). The 1,000,000 *t*-values formed the H1 distributions of between-group differences. Nonparametric p values were calculated as the proportion of permutations that generated *t*-values failing to reach significance (parametric p > 0.05), divided by 1,000,000. Across these variables, the S^+^ group significantly differed from the S^−^ group (ps < 0.038).

## Data Availability

The data supporting the findings of this study are publicly available in the GitHub repository at https://github.com/ZhihaoWangpsyer/elife_suicide (Wang, 2026).

## Additional files

MDAR checklist

## Appendix 1

### Sample characteristics

There was a lack of consensual definition for suicidal thoughts and behaviors (STB; Jollant et al., 2023). See Goodfellow et al., 2018 for a systematic review for nomenclatures of suicidology. In the current study, the threshold for suicidal ideation was active thoughts of suicide, i.e., wishing to die and having some intention to do so, while a suicide attempt was characterized by a deliberate action taken to end one’s life. Consistent with prior suicidal research (Glenn et al., 2019; Miller et al., 2024; Millner et al., 2019), STB referred to individuals either with suicidal ideation or a suicidal attempt. STB has been understood as a transdiagnostic symptom. Indeed, literature reported that STB co-occurred with many mental disorders, such as major depressive disorders (MDD), generalized anxiety disorders (GAD), bipolar disorders (BD), schizophrenia (Pompili et al., 2007), borderline personality disorders (Tsypes et al., 2024), and substance use (Poorolajal et al., 2016), amongst others. Given that many intervention studies of STB mainly focused on mood and anxiety disorders (see Jollant et al., 2023 for a review) and adolescence is an important time window for the emergence of affective problems (O’Connor and Nock, 2014), we thus recruited adolescent patients who were currently diagnosed with MDD, GAD, or BD. Patients were diagnosed by two experienced psychiatrists using the Structured Clinical Interview for DSM-IV-TR-Patient Edition (SCID-P, 2/2001 revision). It is important to note there was no adolescent version of SCID. Therefore, experienced psychiatrists made diagnostic decisions with appropriate adjustments for adolescent populations. Despite not conducting a structured interview to directly assess suicidal risk, the psychiatrists remained vigilant for signs of suicidal ideation and inquired about suicidal-related thoughts and behaviors from both the patients and their families. If the patient openly discussed suicide, the doctor asked follow-up questions, like ‘Have you thought about how you would harm yourself?’ or ‘do you have a specific plan in mind?’. Instead, if the patient did not bring up suicide, the psychiatrist could gently probe with questions, such as ‘Have you been feeling overwhelmed or hopeless lately?’ or ‘Do you ever feel like life is no longer worth living’ to assess potential suicidal thoughts and behaviors.

**Appendix 1—table 1.** A short summary for risk measurement in STB. Abbreviations: BIS, Barratt Impulsiveness Scale; IGT, Iowa Gambling Task; CGT, Cambridge Gambling Task; BART, the Balloon analog risk task.

Study	Measurements	Tools	Analysis level	Hypotheses	Results	Category	Model specification
Millner et al., 2020	Questionnaire	UPPS-P Impulsive Behavior Scale + BIS	Sum (sub)scale scores	Heightened impulsiveness in STB	ns	Self-report	
Zakowicz et al., 2021	Questionnaire	BIS	Sum (sub)scale scores	Heightened impulsiveness in STB	ns	Self-report	
Jollant et al., 2005	Task	IGT	Model-agnostic	More risky behavior in STB	Task performance: STB < control	Risk +Ambiguity + Learning	
Bridge et al., 2012	Task	IGT	Model-agnostic	More risky behavior in STB	Task performance: STB < control	Risk +Ambiguity + Learning	
Martino et al., 2011	Task	IGT	Model-agnostic	More risky behavior in STB	Task performance: STB < control	Risk +Ambiguity + Learning	
Chamberlain et al., 2013	Task	CGT	Model-agnostic	More risky (irrational) behavior in STB	Proportion of rational choices: STB < control	Risk	
Ackerman et al., 2015	Task	CGT	Model-agnostic	More risky behavior in STB	Proportion of bet: STB > control	Risk	
Dir et al., 2020	Task	BART	Model-agnostic	More risky behavior in STB	Task performance: STB < control	Risk +Ambiguity + Learning	
Liu et al., 2022	Task	BART	Model-based	Decision-making bias in STB	Task performance: STB > control Loss aversion: STB > control	Risk +Ambiguity + Learning	Exponential‐Weight Model: loss aversion, risk preference, updating exponent, prior belief of exploding
Baek et al., 2017-risk	Task	Gambling (gain + loss)	Model-based	Heightened risk aversion in STB	Risk aversion: STB > control	Risk	Risk discount model: discount parameter
Baek et al., 2017-loss	Task	Gambling (mix)	Model-based	Heightened loss aversion in STB	Loss aversion: STB > control	risk	Psychophysics; indifference point
Alacreu-Crespo et al., 2020	Task	IGT	Model-based	More risky behavior in STB	Task performance: STB < control; Loss aversion: STB < control; Learning: STB > control	Risk +Ambiguity + Learning	Prospect valence learning delta model: learning/memory, choice consistency, loss aversion, and feedback sensibility
The current study	Task	Gambling (gain + loss + mix)	Model-based	More risky behavior in STB	Gambling behavior: STB > control; Approach parameter: STB > control	Risk	The Approach-Avoidance Prospect Theory Model: risk aversion, loss aversion, approach motivation, avoidance motivation, decision noise

**Appendix 1—table 2.** Contrasts for demographic and psychological characteristics.

	S^−^ vs. HC		S^+^ vs. HC	
	*t*/*χ*^2^	p	*t*/*χ*^2^	p
Gender	0.002	0.967	0.880	0.348
Age	0.816	0.416	–1.458	0.147
BSI-C	2.030	0.044	19.889	<0.001
BSI-W	0.342	0.733	23.723	<0.001
CTQ	3.426	<0.001	8.822	<0.001
ERQ-R	–1.609	0.110	–8.278	<0.001
ERQ-S	2.409	0.017	6.650	<0.001
TAI	3.174	0.002	15.653	<0.001
PSWQ	2.142	0.034	13.364	<0.001
BDI	3.299	0.001	17.368	<0.001
CESD	3.522	<0.001	16.255	<0.001

**Appendix 1—table 3.** Sample size for each diagnosis with and without comorbidity in S^+^ and S^−^ groups.

Diagnosis	S^+^	S^−^	Statistics
GAD	1	2	χ^2^ = 4.843 p = 0.304
MDD	25	9	
BD	7	6	
MDD and BD	2	0	
MDD and AD	23	8	

**Appendix 1—table 4.** Bivariate correlations between choice parameters and socio-demographic clinical variables. All p values were above 0.05.

		*λ*	*α*	*β*_gain_	*β*_loss_	*μ*
**Demographics**	**Gender**	rho = 0.00, p = 1.000	rho = 0.15, p = 0.179	rho = 0.08, p = 0.495	rho = 0.14, p = 0.197	rho = –0.00, p = 0.961
	**Age**	rho = 0.13, p = 0.230	rho = 0.07, p = 0.517	rho = –0.19, p = 0.079	rho = –0.03, p = 0.811	rho = –0.17, p = 0.114
**Social variables**	**CTQ**	rho = 0.01, p = 0.946	rho = –0.19, p = 0.090	rho = 0.08, p = 0.495	rho = 0.04, p = 0.700	rho = 0.20, p = 0.075
	**ERQ-E**	rho = 0.14, p = 0.215	rho = –0.11, p = 0.338	rho = –0.17, p = 0.114	rho = –0.21, p = 0.059	rho = –0.03, p = 0.769
	**ERQ-S**	rho = –0.13, p = 0.230	rho = –0.04, p = 0.727	rho = 0.15, p = 0.187	rho = 0.10, p = 0.346	rho = 0.17, p = 0.128
**Clinical variables**	**Illness duration**	rho = 0.08, p = 0.480	rho = –0.04, p = 0.737	rho = –0.05, p = 0.650	rho = –0.02, p = 0.849	rho = –0.10, p = 0.351
	**Family history**	rho = 0.16, p = 0.142	rho = 0.04, p = 0.706	rho = 0.05, p = 0.648	rho = –0.04, p = 0.700	rho = –0.01, p = 0.908
	**MDD**	rho = –0.11, p = 0.317	rho = 0.17, p = 0.123	rho = 0.02, p = 0.882	rho = –0.04, p = 0.697	rho = –0.11, p = 0.324
	**GAD**	rho = 0.11, p = 0.335	rho = 0.16, p = 0.162	rho = 0.02, p = 0.851	rho = 0.03, p = 0.797	rho = –0.03, p = 0.755
	**BD**	rho = 0.17, p = 0.124	rho = –0.12, p = 0.288	rho = 0.03, p = 0.801	rho = 0.04, p = 0.707	rho = 0.05, p = 0.630

**Appendix 1—table 5.** Bivariate correlations between mood parameters and socio-demographic clinical variables. p values lower than 0.05 were highlighted in bold.

		*β*_CR_	*β*_GR_	*γ*	*β*_o_
**Demographics**	**Gender**	rho = 0.15, p = 0.168	rho = 0.23, p = 0.033	rho = 0.08, p = 0.465	rho = –0.17, p = 0.126
	**Age**	rho = 0.07, p = 0.503	**rho = –0.27,** **p = 0.012**	rho = 0.06, p = 0.615	**rho = 0.28,** **p = 0.010**
**Social variables**	**CTQ**	rho = 0.04, p = 0.739	rho = 0.07, p = 0.545	rho = –0.06, p = 0.610	rho = –0.02, p = 0.866
	**ERQ-E**	rho = 0.09, p = 0.440	**rho = –0.26,** **p = 0.017**	rho = –0.13, p = 0.255	**rho = 0.46,** ***P* < 0.001**
	**ERQ-S**	rho = –0.19, p = 0. 086	rho = –0.06, p = 0.59 9	rho = –0.14, p = 0.217	rho = –0.16, p = 0.142
**Clinical variables**	**Illness duration**	rho = –0.01, p = 0.910	rho = –0.10, p = 0.366	rho = –0.03, p = 0.788	rho = –0.02, p = 0.861
	**Family history**	rho = 0.06, p = 0.590	rho = –0.01, p = 0.908	rho = –0.09, p = 0.403	rho = 0.13, p = 0.231
	**MDD**	rho = –0.02, p = 0.864	rho = 0.09, p = 0.396	rho = –0.07, p = 0.521	rho = –0.21, p = 0.053
	**GAD**	rho = –0.06, p = 0.569	rho = 0.05, p = 0.627	rho = 0.01, p = 0.912	rho = –0.08, p = 0.491
	**BD**	rho = 0.16, p = 0.153	rho = –0.07, p = 0.542	rho = –0.02, p = 0.888	rho = 0.16, p = 0.160

**Appendix 1—table 6.** Mood model comparison by separating gambling outcomes into better and worse parts.

Model #	Model specification	# of parameters	Δ BIC	Mean *R*^2^	Δ BIC for each group		
					HC	S^−^	S^+^
**mM3**	***β*_0_, *β*_CR_, *β*_GR_, *γ***	**4**	**0**	**0.42**	**0**	**0**	**0**
mM7	*β*_0_, *β*_CR_, *β*_GR_better_, *β*_GR_worse_, *γ*	5	**–331.48**	0.49	**–355.10**	**–6.56**	30.18
mM8	*β*_0_, *β*_CR_, *β*_GR_better_, *β*_GR_worse_, *γ*_CR_, *γ*_GR_better_, *γ*_GR_better_	7	–105.40	0.56	–313.09	81.34	126.34

^Δ^BIC, Bayesian information criterion relative to the winning model in S^+^ group (mM3); HC, healthy control; S^-^, patients without suicidal thoughts and behavior; S^+^, patients with suicidal thoughts and behavior.

**Appendix 1—table 7.** Choice model comparison by integrating mood or adding traditional bias.

Model #	Model specification	# of parameters	Δ BIC	Mean *R*^2^	Δ BIC for each group		
					HC	S^−^	S^+^
**cM3**	***λ*, *α*, *β*_gain_, *β*_loss_, *µ***	**5**	**0**	**0.37**	**0**	**0**	**0**
cmM1	*λ*, *α*, *β*_gain_, *β*_loss_, *µ*, *β*_Mood_	6	5530.23	0.39	287.20	68.76	174.27
cmM2	*λ*, *α*, *β*_gain_, *β*_loss_, *µ*, β_Mood-CR,_ β_Mood-GR_	7	1031.18	0.41	577.03	123.84	330.32
cM4	***λ*, *α*, *µ*, *β*_bias_**	4	376.75	0.32	277.65	34.31	64.79

^Δ^BIC, Bayesian information criterion relative to the winning model (cM3); HC, healthy control; S^-^, patients without suicidal thoughts and behavior; S^+^, patients with suicidal thoughts and behavior.

**Appendix 1—table 8.** Mood model comparison by adding a term for whether participants gambled or not, independent of the gambling value.

Model #	Model specification	# of parameters	Δ BIC	Mean *R*^2^	Δ BIC for each group		
					HC	S^-^	S^+^
**mM3**	***β*_0_, *β*_CR_, *β*_GR_, γ**	**4**	**0**	**0.42**	**0**	**0**	**0**
mM9	*β*_0_, *β*_CR_, *β*_GR_ *β*_gamble_, *γ*	5	**–373.13**	0.49	**–371.83**	**–40.74**	39.45

^Δ^BIC, Bayesian information criterion relative to the winning model in S^+^ group (mM3); HC, healthy control; S^-^, patients without suicidal thoughts and behavior; S^+^, patients with suicidal thoughts and behavior.

**Appendix 1—table 9.** Bayesian independent sample t-tests of median-split anxiety and depression scores (including TAI, PSWQ, BDI, and CESD) on main results (gambling rate, approach parameter (*β*_gain_), and mood sensitivity to certain rewards (***β*_CR_**)) support that general symptoms of anxiety and depression overall did not influence our main results. BF₀₁ is a Bayes factor comparing the null model (M₀) to the alternative model (M₁), where M₀ assumes no group difference. BF₀₁ >1 indicates that evidence favors M₀. Generally, Bayes Factors between 1 and 3 were interpreted as anecdotal evidence and between 3 and 10 as moderate evidence.

BF_01_	TAI	PSWQ	BDI	CESD
**Gambling rate**	4.080	2.768	2.425	0.820
***β*_gain_**	3.819	3.911	2.987	1.128
***β*_CR_**	3.704	3.826	1.185	3.178

**Appendix 1—table 10.** Linear regressions of gambling behavior, value-insensitive approach parameter (***β*_gain_**), and mood sensitivity to certain rewards (***β*_CR_**) on group as a predictor (1 for S^+^ group and 0 for S^-^ group) and scores for anxiety and depression as covariates.

	Gambling rate	*β*_gain_	*β*_CR_
**Group**	***β* = 0.164, t = 2.305,** **p = 0.024**	***β* = 0.374, t = 2.257,** **p = 0.027**	***β* = –0.105, t = –3.461,** **p = 0.001**
**TAI**	*β* = –0.010, t = –1.796, p = 0.077	*β* = –0.008, t = –0.649, p = 0.519	*β* = 0.005, t = 1.921, p = 0.059
**PSWQ**	*β* = –0.001, t = –0.401, p = 0.690	*β* = –0.008, t = –0.968, p = 0.337	*β* = –0.002, t = –1.282, p = 0.204
**BDI**	*β* = 0.002, t = 0.519, p = 0.606	*β* = –0.003, t = –0.272, p = 0.787	***β* = –0.004, t = –2.302,** **p = 0.025**
**CESD**	*β* = 0.006, t = 1.585, p = 0.118	*β* = 0.015, t = 1.652, p = 0.103	*β* = 0.003, t = 1.942, p = 0.056

**Appendix 1—table 11.** Linear regressions of gambling behavior, value-insensitive approach parameter (***β*_gain_**), and mood sensitivity to certain rewards (***β*_CR_**) on group as a predictor (1 for S^+^ group and 0 for S^-^ group) and orthogonal components of anxiety and depression as covariates.

	Gambling rate	*β*_gain_	*β*_CR_
**Group**	***β* = 0.164, t = 2.305,** **p = 0.024**	***β* = 0.378, t = 2.257,** **p = 0.027**	***β* = –0.105, t = –3.461,** **p = 0.001**
**PC1**	*β* = –0.007, t = –0.425, p = 0.672	*β* = –0.010, t = –0.247, p = 0.806	*β* = 0.009, t = 1.169, p = 0.247
**PC2**	*β* = –0.073, t = –1.569, p = 0.122	*β* = –0.164, t = –1.509, p = 0.136	*β* = –0.011, t = –0.532, p = 0.596
**PC3**	*β* = 0.098, t = 1.429, p = 0.158	*β* = 0.205, t = 1.283, p = 0.204	*β* = 0.037, t = 1.255, p = 0.214
**PC4**	*β* = –0.086, t = –1.133, p = 0.262	*β* = 0.010, t = 0.054, p = 0.957	***β* = 0.086, t = 2.657,** **p = 0.010**

## Appendix 2

### Suicidal attempts vs. suicidal ideations

Consistent with previous suicidal-related literature (Eisenlohr-Moul et al., 2018; Glenn et al., 2019; Glenn et al., 2017; Miller et al., 2017; Miller et al., 2024; Millner et al., 2019), this study focused on why adolescent patients with STB showed more risky behavior. By dividing patients into two groups: patients with STB (S^+^) and without STB (S^-^), this work revealed the general tendency for suicidal risks and had clinical implications for suicidal prevention, especially among adolescents. Although not at the heart of this study, as a secondary analysis, we checked differences in patients with suicidal attempts (SA) and without SA. To carefully control for suicidal ideations (SI), we mainly checked differences between SA and suicidal ideation (SI) groups. However, results showed no significant group difference in either choice or mood indices (ps > 0.148; Appendix 1—figure 1).

## Appendix 3

### Sensitivity analysis for suicidal timeframe

In this study, we focused on adolescents (age: 10–19 years) who made suicidal thoughts and behaviors during their adolescent period (10–19 years). The longest timeframe for STB was 6 years (mean: 14.6 months; median: 6 months; standard deviation: 17.74). If we limited the timeframe, e.g., midpoint of the longest timeframe (3 years; 36 months), all results, including increased risky behavior, heightened approach motivation, and lower mood sensitivity to certain reward in STB, remained significant (ps < 0.026).

## Appendix 4

### Control analysis for childhood maltreatment, emotion regulation, and depression/anxiety symptoms

It has been shown that patients with STB have childhood maltreatment problems, inability to regulate their emotions adaptively, and high levels of depression and anxiety per se (Neacsiu et al., 2018; Sarchiapone et al., 2007). Indeed, we observed these patterns in our data (see Table 1). To check whether our current effects were specific to suicidal thoughts and behaviors, but not these symptoms, we performed control analysis for these variables. Specifically, we used median split to check each potential confound on the gambling chosen, approach parameter, and mood sensitivity to certain reward. Results showed no significant effect in the variables of interest (ps > 0.056; Appendix 1—figures 3 and 4). In addition, our recent work identified the specific influence of depression/anxiety on mood sensitivity to reward prediction error, but not mood sensitivity to certain reward (Wang et al., 2025), further suggesting that the current group differences were specific to suicidal thoughts and behaviors, but not general severity of internalizing psychopathology.

## Appendix 5

### Justify for the modeling technique

#### Model recovery

We performed simulation-based model recovery analyses over 1,000 iterations. In each iteration, the task design matrix was randomly shuffled to vary trial order while maintaining the original task structure. For choice model recovery, for each of the three candidate choice models, a random parameter set was generated and used to simulate choices based on the shuffled design matrix. Each simulated dataset was then fit with all three choice models, and BIC was computed for each fit. The model with the lowest BIC was selected as the winning model. Recovery performance was summarized in a confusion matrix, where each cell reflected the proportion of times a given model was selected when data were generated from a particular model.

For mood model recovery, simulated outcomes were first generated from the fitted choice model and then used to simulate mood ratings. For each of the six candidate mood models, random parameters were generated and used to produce synthetic mood data. Mood model recovery was performed in a stage-by-stage manner, mirroring the model comparison procedure used in the main analyses because each model comparison step addresses a specific question and avoids dispersion of model evidence across multiple similar models within over-represented model families, which can reduce apparent recovery of the true generative model. Specifically, rather than entering all mood models into a single recovery space, recovery was evaluated separately within each comparison space used for model selection. Thus, each confusion matrix reflects recovery among only the models that were directly compared at that stage. Note that the winning model was determined at the individual level. As shown in Appendix 1—figure 5, model identifiability was high overall, indicating good recovery performance for both the choice and mood models.

#### Parameter recovery

To assess parameter recovery, the generating parameters were further correlated with the recovered parameters from the corresponding fitted model. Note that simulated datasets were not generated from parameters estimated from empirical data. Instead, parameter values were randomly drawn from the predefined bounded parameter space used for model fitting. This approach was adopted to minimize the correlation structure inherited from the empirical fits and to promote greater orthogonality among parameters, thereby providing a cleaner test of parameter recoverability. Appendix 1—figure 6 shows good parameter recovery for both choice and mood winning model (choice: rs > 0.37, ps < 0.001; intraclass coefficients >0.24; mood: *r*s > 0.99, ps <0.001; intraclass coefficients > 0.99). Moreover, we computed cross-correlations between all generating (‘generating’) and recovered (‘fitted’) parameters. The resulting matrix showed high diagonal (choice winning model: *r*s > 0.37; mood winning model: *r*s > 0.99) and low off-diagonal (choice winning model: abs(*r*s) < 0.11; mood winning model: abs(*r*s) < 0.08) correlations, further supporting parameter recovery.

#### Model fitting approach

It is true that hierarchical Bayesian estimation and Markov chain Monte Carlo (MCMC) are promising approaches for model fitting (Ahn et al., 2017; Piray et al., 2019). However, the maximum likelihood-based model fitting procedure (MLE), e.g., fmincon in Matlab, was widely used, especially in domains of risky decision making and mood science (Blain and Rutledge, 2020; Rutledge et al., 2014), with the advantage of less time cost. Importantly, recent literature shows the same pattern between the two approaches (Rutledge et al., 2014; Vanhasbroeck et al., 2021). Specifically, Vanhasbroeck et al., 2021 reproduced all results in a hierarchical way with those in the MLE way in the context of gambling with momentary mood ratings (the same as the current task), suggesting the reliability of MLE, at least for the current task. Excellent performance of parameter recovery and model recovery also demonstrated that MLE in this task was reliable.

#### Parameter range

The boundaries of parameter ranges were the same as previous literature (Blain and Rutledge, 2020; Rutledge et al., 2014). These boundaries showed weakly informative priors. Take an example of loss aversion ($\mathrm{\lambda }$: 0.5–5). The value of $\mathrm{\lambda }$ higher than 1 suggests loss aversion, while lower than 1 suggests loss seeking. The higher the $\mathrm{\lambda }$, the more loss aversion. This boundary allowed a flexible feature for loss attitude. Based on classic prospect theory, people, on average, are aversive to loss. Therefore, there was more space for loss aversion, while less space for loss seeking.

#### *R* ^2^

Although *R*^2^ for continuous data or pseudoR^2^ for dichotomous data is an important fitting matrix, e.g., assessment for fitting performance, limited research reported it. Based on the existing literature, pseudoR^2^ of 0.37 for the choice winning model was comparable to the previous literature using the same task: 0.46, lower than that using the simple tasks (0.86 for effort-based decision-making task Lockwood et al., 2021) and 0.54 for reinforcement learning task (Blain and Rutledge, 2020), but higher than that using more complex tasks (pseudo *R*^2^ of 0.26 for two-step task [Daw et al., 2011]). For mood models, *R*^2^ of 0.42 was comparable to previous mood models (Blain and Rutledge, 2020; Rutledge et al., 2014).

## Appendix 6

### Integration of mood into choice models

Although we modeled choice and mood separately to examine cognitive and affective mechanisms underlying increased risk behavior in adolescent suicidal patients, one interesting question was whether mood responses influence subsequent gambling choices and how to model them. First, we median-split mood responses (except the final rating) to compare gambling rate. Results showed a trend for less gambling rate in higher mood (*t*=–1.971, *P*=0.050). However, there was no significant group difference (*F*=0.680, *P*=0.507). Second, with the assumption that mood biases choice, we constructed mcM1 based on cM3 (the winning choice model).

(12) $$\displaystyle P_{gamble}={\, }\frac{1-\beta _{val}}{1+e^{-\mu \left (U_{gamble}-U_{certain}+\beta _{Mood}\ zMood\right)}}+\beta _{val}{\, }{\, }if{\, }\beta _{val}\geq 0$$

(13) $$\displaystyle P_{gamble}={\, }\frac{1+\beta _{val}}{1+e^{-\mu \left (U_{gamble}-U_{certain\ +\beta _{Mood}\ zMood}\right)}}{\, \, }if{\, }\beta _{val}< 0{\, }$$

(14) $$\displaystyle \beta _{val}\left \{\begin{array}{c}\beta _{gain},\, \, gain\, trials,\\\beta _{loss},\, \, loss\, trials.\end{array}\right.$$

Based on our finding of the negative correlation between mood sensitivity to certain rewards and gambling rate in S^+^, we separated β_Mood_ parameter into β_Mood-CR_ and β_Mood-GR_ (cmM2).

(15) $$\displaystyle \beta _{Mood}\left \{\begin{array}{c}\beta _{Mood-CR},\, \, certain\, choices,\\\beta _{Mood-GR},\, \, gamble\, choices.\end{array}\right.$$

Model comparison using BIC supported cM3 (Appendix 1—table 7), that is, without consideration of mood in choice modeling. The mood bias parameters in neither cM2 nor cM3 reached significance (ps >0.091), which may be due to the absence of a blocked design in our experiment, unlike in Vinckier et al., 2018 and Eldar et al., 2016.

## Appendix 7

### Other candidate models

We also considered the traditional bias parameter (cM4), rather than approach/avoidance parameters. We limited the bias to the range of [–100, 100], which was in reward-equivalent units.

(16) $$\displaystyle P_{gamble}={\, }\frac{1}{1+e^{-\mu \left (U_{gamble}-U_{certain}+\beta _{bias}\, \right)}}$$

However, model comparison did not support cM4 (Appendix 1—table 7).

For the mood models, we additionally considered a term capturing whether participants gambled or not, independent of the gambling value (mM9), based on the winning model mM3. Given that the primary focus of the current study was the effect of STB, particularly in the S^+^ group, using the S^−^ and HC groups as baseline controls, we focused primarily on the winning model identified in the S^+^ group. Model comparison supported mM3 as the winning model in the S^+^ group (Appendix 1—table 8). Nevertheless, we also examined the results obtained with mM9. Overall, this additional analysis replicated the significant group differences in mood sensitivity to CR, both between S^+^ and S^−^ (*t*=–5.480, *P*=0.015) and between S^+^ and HC (*t*=–2.025, *P*=0.044), whereas S^−^ and HC did not differ significantly from each other (*t*=0.628, *P*=0.531). By contrast, no significant group differences were found in mood sensitivity to GR or in mood sensitivity to gambling (ps >0.121).

## Appendix 8

### Clarification for FDR correction

In the clinical dataset we conducted a large number of inferential tests (*χ*², *t*-tests, ANOVAs, regressions) spanning: (1) group differences in demographic/clinical characteristics; (2) sanity checks (e.g., anxiety/depression questionnaires); (3) primary hypotheses (e.g., group differences in risky behavior); (4) model-based analyses (parameter checks and between-group contrasts); and (5) control/sensitivity analyses. Post hoc *t*-tests were performed only when the three-group ANOVA was significant. This yielded >150 p values. FDR was applied using all these p values.

## Appendix 9

### Mood model comparison using subjective values

To identify whether mood modeling was based on objective or subjective values, we constructed two model families: one in which mood was driven by objective monetary outcomes (objective values) and one in which mood was driven by subjective values derived from each participant’s fitted choice model (subjective values). We then used the VBA_groupBMC function in the VBA toolbox (Daunizeau et al., 2014) to perform family-wise model comparison, with 6 candidate mood models within each family. Consistent with previous literature, the objective-value family provided a clearly superior fit to the data (exceedance probability, EP = 1.000).
