# Supplementary material for: Mood computational mechanisms underlying increased risk behavior in adolescent suicidal patients
Source: eLife. 2026 Aug 7;14:RP108002. doi: 10.7554/eLife.108002 (PMC13451020; doi:10.7554/eLife.108002)
Supplement: Peer review history

# Editor report: eLife Assessment

**DOI:** 10.7554/eLife.108002.4.sa0

**Authors:** Jonathan Roiser

**Affiliations:** University College London United Kingdom

**Keywords:** Convincing, Important

This **important** study combined careful computational modeling, a large patient sample, and replication in an independent general population sample to provide **convincing** evidence in support of a computational account of a difference in risk-taking between people who have attempted suicide and those who have not. It is proposed that this difference reflects a general change in the approach to risky (high-reward) options and a lower emotional response to certain rewards. While the findings advance our understanding of cognitive mechanisms at the group level, the observation that computational phenotype is predictive of suicidal behavior only in the clinical sample and not in the online sample limits its applicability for individual prediction, early detection and prevention of suicidality.

# Referee report: Reviewer #1 (Public review)

**DOI:** 10.7554/eLife.108002.4.sa1

Summary:

The authors use a gambling task with momentary mood ratings from Rutledge et al. and compare computational models of choice and mood to identify markers of decisional and affective impairments underlying risk-prone behavior in adolescents with suicidal thoughts and behaviors (STB). The results show that adolescents with STB show enhanced gambling behavior (choosing the gamble rather than the sure amount), and this is driven by a bias towards the largest possible win rather than insensitivity to possible losses. Moreover, this group shows a diminished effect of receiving a certain reward (in the non-gambling trials) on mood. The results were replicated in a general online sample where participants were divided into groups with or without STB based on their self-report of suicidal ideation on one question in the Beck Depression Inventory self-report instrument. The authors suggest, therefore, that adolescents diagnosed with depression or anxiety with decreased sensitivity to certain rewards may need to be monitored more closely for STB due to their increased propensity to take risky decisions aimed at (expected) gains (such as relief from an unbearable situation through suicide) regardless of the potential losses. However, such a result was only found in the clinical sample and cannot be generalized more broadly based on the current findings.

Strengths:

● The study uses a previously validated task design and replicates previously found results through well-explained model-free and model-based analyses.

● Sampling of adolescents at high risk can help target early preventative diagnoses and treatments for suicide.

● Replication of the results in an online cohort increases confidence in the findings.

● The models considered for comparison are thorough and well-motivated. The chosen models allow for teasing apart which decision and mood sensitivity parameters relate to risky decision-making across groups based on their hypotheses.

● Novel finding of mood (in)sensitivity to non-risky rewards and its relationship with risk behavior in STB.

Weaknesses:

● Sample size of 25 for S- group is low-powered, which is explicitly mentioned as a study limitation.

● Modeling in the mediation analysis focused on predicting risk behavior in this task from the model-derived bias for gains and suicidal symptom scores. Thus, the implications of this work are more relevant to a basic-science understanding of the etiology of suicidal behavior than they are useful as a predictor of suicidal behavior, and it is not clear that a psychiatrist or psychologist could use this task to potentially determine who is at higher risk of attempting suicide and must be more closely monitored. Indeed, relationships between task parameters and behavior and suicidal behavior was limited to the clinical sample with a diagnosis of depression or anxiety disorder, and did not extend to the online sample. Therefore, the claim that these findings provide "computational markers for general suicidal tendency among adolescents" is unwarranted.

# Referee report: Reviewer #2 (Public review)

**DOI:** 10.7554/eLife.108002.4.sa2

Summary:

This article addresses a very pertinent question - what are the computational mechanisms underlying risky behaviour in patients having attempted suicide. In particular, it is impressive how the authors find a broad behavioral effect whose mechanisms they can then explain and refine through computational modeling. This work is important because currently, beyond previous suicide attempts, there has been a lack of predictive measures. This study is the first step towards that: understanding the cognition on a group level. Before then being able to include it in future predictive studies (based on the cross-sectional data, this study by itself cannot assess the predictive validity of the measure).

Strengths:

- Large sample size

- Replication of their own findings

- Well-controlled task with measures of behaviour and mood + precise and well-validated computational modeling

Questions, based on revised manuscript and replies to other reviewers:

(1) Replies to reviewers in general: Bayes Factors have been added, it would be good to also use common verbal terms to describe them (e.g. 'anecdotal', 'moderate' etc). For example, my reading of table S8 would be that for gambling rate there is only anecdotal evidence that it does not relate to PSWQ, BDI, and moderate evidence it does not relate to TAI.

(2) Reply to reviewer 1 Q2 (Predicting STB):

For the regression predicting suicidal ideation, it seems to me that what you did was a regression STB ~ gambling behaviour + approach + mood? Could you clarify? I had expected as a test of whether the task can predict STB risk something slightly different - a cross-validation (LOO or maybe 5-fold in the large sample): STB ~ gambling behaviour + approach [parameter from model] + mood [parameter from model]; and then computing in the left out participants: predicted STB. Then checking correlation between STB and predicted STB. This would allow testing whether the diverse task measures together predict STB (with the caveat, that it's cross-validated, rather than hold-out sample, unless you could train on one sample (in lab) and test on the other (online)).

(3) Reply to reviewer 2 Q1 (parameter recovery): I'm looking at S3, it seems to still show only the scatter plots and not the correlation matrices, which are now added as text notes. Can you actually show these matrices? An off-diagonal correlation of 0.63 appears quite high. I think it needs to be discussed exactly which parameters those are, and whether that impacts the interpretation of the results.

(4) Reply to reviewer 3 Q3 (mood model): I would have imagined that the response would involve changing the mood equations (equation 8 main text) to include a term for whether the participant gambled or not, independent of the gamble value.

# Referee report: Reviewer #3 (Public review)

**DOI:** 10.7554/eLife.108002.4.sa3

This manuscript investigates computational mechanisms underlying increased risk-taking behavior in adolescent patients with suicidal thoughts and behaviors. Using a well-established gambling task that incorporates momentary mood ratings and previously established computational modeling approaches, the authors identify particular aspects of choice behavior (which they term approach bias) and mood responsivity (to certain rewards) that differ as a function of suicidality. The authors replicate their findings on both clinical and large-scale non-clinical samples.

The main problem, however, is that the results do not seem to support a specific conclusion with regard to suicidality. The S+ and S- groups differ substantially in the severity of symptoms, as can be seen by all symptom questionnaires and the baseline and mean mood, where S- is closer to HC than it is to S+. The main analyses control for illness duration and medication but not for symptom severity. The supplementary analysis in Figure S11 is insufficient as it mistakes the absence of evidence (i.e., p > 0.05) for evidence of absence. Therefore, the results do not adequately deconfound suicidality from general symptom severity.

The second main issue is that the relationship between an increased approach bias and decreased mood response to CR is conceptually unclear. In this respect, it would be natural to test whether mood responses influence subsequent gambling choices. This could be done either within the model by having mood moderate the approach bias or outside the model using model-agnostic analyses.

Additionally, there is a conceptual inconsistency between the choice and mood findings that partly results from the analytic strategy. The approach bias is implemented in choice as a categorical value-independent effect, whereas the mood responses always scale linearly with the magnitude of outcomes. One way to make the models more conceptually related would be to include a categorical value-independent mood response to choosing to gamble/not to gamble.

The manuscript requires editing to improve clarity and precision. The use of terms such as "mood" and "approach motivation" is often inaccurate or not sufficiently specific. There are also many grammatical errors throughout the text.

Claims of clinical relevance should be toned down, given that the findings are based on noisy parameter estimates whose clinical utility for the treatment of an individual patient is doubtful at best.

Comments on revisions:'

The authors adequately addressed my comments and I find the manuscript substantially strengthened.

# Author comment: Author response

**DOI:** 10.7554/eLife.108002.4.sa4

**Authors:** Zhihao Wang, Tian Nan, Fengmei Lu, Yu Yue, Xiao Cai, Zongling He, Yuejia Luo, Ting Wang, Bastien Blain

**Affiliations:** Shenzhen University of Advanced Technology Shenzhen China; Beijing Normal University Beijing China; University of Electronic Science and Technology of China Chengdu China; University of Electronic Science and Technology of China Chengdu China; University of Electronic Science and Technology of China Chengdu China; University of Electronic Science and Technology of China Chengdu China; Beijing Normal University Beijing China; South China Normal University Guangzhou China; University College London London United Kingdom

The following is the authors’ response to the previous reviews

**eLife Assessment**

This valuable study combined careful computational modeling, a large patient sample, and replication in an independent general population sample to provide a computational account of a difference in risk-taking between people who have attempted suicide and those who have not. It is proposed that this difference reflects a general change in the approach to risky (high-reward) options and a lower emotional response to certain rewards. Evidence for the specificity of the effect to suicide, however, is incomplete, which would require additional analyses.

We thank the editors and reviewers for this important assessment. Based on clinical interviews, we included patients with and without suicidality (S^+^ and S^-^ groups). However, in line with suicidal-related literature (e.g., Tsypes et al., 2024), two groups also differed substantially in the severity of symptoms (see Table 1). To address the request for evidence on specificity to suicidality beyond general symptom severity, we performed separate linear regressions to explain in gambling behaviour, value-insensitive approach parameter (β_gain_), and mood sensitivity to certain rewards (β_CR_) with group as a predictor (1 for S^+^ group and 0 for S^-^ group) and scores for anxiety and depression as covariates. Results remained significant after controlling anxiety and depression (*ps* < 0.027; Table S8). Given high correlations among anxiety and depression questionnaires (rs > 0.753, *ps* < 0.001), we performed Principal Components Analysis (PCA) on the clinical questionnaire to extract the orthogonal components, where each component explained 86.95%, 7.09%, 3.27%, and 2.68% variance, respectively. We then performed linear regressions using these components as covariates to control for anxiety and depression. Our main results remained significant (*ps* < 0.027; Table S9). We believe that these analyses provide evidence that the main effects on gambling and on mood were specific to suicide.

Moreover, as Reviewer 3 pointed out, these “absence of evidence” cannot provide insights of “evidence of absence”. Although we median-split patients by the scores of general symptoms (e.g., depression and anxiety-related questionnaires) and verified no significant differences in these severities (Figure S11), we additionally conducted Bayesian statistics in gambling behavior, value-insensitive approach parameter, and mood sensitivity to certain rewards. BF_01_ is a Bayes factor comparing the null model (M_0_) to the alternative model (M_1_), where M_0_ assumes no group difference. BF_01_ > 1 indicates that evidence favors M_0_. As can be seen in Table S7, most results supported null hypothesis, suggesting that general symptoms of anxiety and depression overall did not influence our main results. Overall, we believe that these analyses provide compelling evidence for the specificity of the effect to suicide, above and beyond depression and anxiety.

Beyond these specific findings, this work highlights the broader utility of computational modelling and mood to better understand behavioral effect, showing how to use both mood and choice data to better comprehend a psychiatric issue.

Please see Tables S7, S8, S9 and our revisions below:.

Page 17:

“Within patients, this group effect on gambling rate remained significant after controlling for sex, illness duration, family history, diagnosis, and various medications use (*ps* < 0.05), as well as general symptoms (e.g., depression and anxiety; *p* = 0.024; also see Figure S11, Table S7 and Table S8). Given high correlations among anxiety and depression questionnaires (rs > 0.753, *ps* < 0.001), we performed Principal Components Analysis (PCA) to extract main components, where each component explained 86.95%, 7.09%, 3.27%, and 2.68% variance, respectively. To further control for anxiety and depression, linear regression using these components as covariates revealed that the group effect on gambling rate remained significant (*p* = 0.024; Table S9).”

Pages 18-19:

“Within patients, this group effect on the approach parameter remained significant after controlling for sex, illness duration, family history, diagnosis, and various medications use (*ps* < 0.05), as well as general symptoms (e.g., depression and anxiety; *p* = 0.027; also see Figure S11, Table S7 and Table S8). Linear regression using PCA components as covariates revealed that the group effect on approach parameter remained significant (*p* = 0.027; Table S9).”

Page 21:

“Within patients, this group effect on βCR remained significant after controlling for gambling rate, earnings, mood-related outcome effect, mood drift effect, sex, illness duration, family history, diagnosis, and various medications use (ps < 0.032), as well as general symptoms (e.g., depression and anxiety; p = 0.001; also see Figure S11, Table S7 and Table S8). Linear regression using PCA components as covariates revealed that the group effect on this mood parameter remained significant (p = 0.001; Table S9).”

Page 27:

“Beyond these specific findings, this work highlights the broader utility of computational modelling and mood to better understand behavioral effect, showing how to use both mood and choice data to better comprehend a psychiatric issue.”

**Public Reviews:**

**Reviewer #1 (Public review):**

Summary:

The authors use a gambling task with momentary mood ratings from Rutledge et al. and compare computational models of choice and mood to identify markers of decisional and affective impairments underlying risk-prone behavior in adolescents with suicidal thoughts and behaviors (STB). The results show that adolescents with STB show enhanced gambling behavior (choosing the gamble rather than the sure amount), and this is driven by a bias towards the largest possible win rather than insensitivity to possible losses. Moreover, this group shows a diminished effect of receiving a certain reward (in the non-gambling trials) on mood. The results were replicated in an undifferentiated online sample where participants were divided into groups with or without STB based on their self-report of suicidal ideation on one question in the Beck Depression Inventory self-report instrument. The authors suggest, therefore, that adolescents with decreased sensitivity to certain rewards may need to be monitored more closely for STB due to their increased propensity to take risky decisions aimed at (expected) gains (such as relief from an unbearable situation through suicide), regardless of the potential losses.

Strengths:

(1) The study uses a previously validated task design and replicates previously found results through well-explained model-free and model-based analyses.

(2) Sampling choice is optimal, with adolescents at high risk; an ideal cohort to target early preventative diagnoses and treatments for suicide.

(3) Replication of the results in an online cohort increases confidence in the findings.

(4) The models considered for comparison are thorough and well-motivated. The chosen models allow for teasing apart which decision and mood sensitivity parameters relate to risky decision-making across groups based on their hypotheses.

(5) Novel finding of mood (in)sensitivity to non-risky rewards and its relationship with risk behavior in STB.

Weaknesses:

(1) The sample size of 25 for the S- group was justified based on previous studies (lines 181-183); however, all three papers cited mention that their sample was low powered as a study limitation.

We thank the Reviewer for rising this concern. We agree that the sample size for S^-^ group (n=25) is modest, and the prior studies we cited also acknowledged limited power. We wanted to point out that we obtained a comparable sample size to a prior study. In the revision, we therefore updated the section to justify this sample size in which we acknowledge the limited power of our study in the limitation section. Please see our clarification below:

Page 32:

“Third, despite replicating our main results in an independent dataset (n=747), the modest S^-^ subgroup size (n=25) has a limited statistical power.”

(2) Modeling in the mediation analysis focused on predicting risk behavior in this task from the model-derived bias for gains and suicidal symptom scores. However, the prediction of clinical interest is of suicidal behaviors from task parameters/behavior - as a psychiatrist or psychologist, I would want to use this task to potentially determine who is at higher risk of attempting suicide and therefore needs to be more closely watched rather than the other way around (predicting behavior in the task from their symptom profile). Unfortunately, the analyses presented do not show that this prediction can be made using the current task. I was left wondering: is there a correlation between beta_gain and STB? It is also important to test for the same relationships between task parameters and behavior in the healthy control group, or to clarify that the recommendations for potential clinical relevance of these findings apply exclusively to people with a diagnosis of depression or anxiety disorder. Indeed, in line 672, the authors claim their results provide "computational markers for general suicidal tendency among adolescents", but this was not shown here, as there were no models predicting STB within patient groups or across patients and healthy controls.

Thank you for these thoughtful comments. Our study focuses on why adolescent patients with suicidality have increased risk behavior, aiming to provide a mechanism-based target for suicide prevention. Therefore, our dependent variable in the mediation model was gambling behavior. We also agree that the clinically relevant question is whether suicidality can be predicted from task-derived behavior/parameters. We thus used risky behavior and the potential mental parameters to predict STB. Linear regressions showed that gambling behavior, as well as the value-insensitive approach parameter, can predict suicidal symptom scores among patients (former: β = 9.189, t = 2.004, *p* = 0.048; latter: β = 5.587, t = 2.890, *p* = 0.005). In healthy controls, these predictions failed (gambling behavior: β = 1.471, t = 0.825, *p* = 0.411; approach: β = 0.874, t = 1.178, *p* = 0.241). These results suggest that clinical relevance of these findings apply exclusively to people with a diagnosis of depression or anxiety disorder. We found same patterns for the mood parameter (mood sensitivity to certain rewards: patients: β = -28.706, t = -2.801, *p* = 0.006; healthy controls: β = -2.204, t = -0.528, *p* = 0.599). In sum, we believe that our statement of “computational markers for general suicidal tendency among adolescents” is reasonable now. Please see our revisions below:

Page 17:

“Furthermore, linear regression showed that gambling rate can predict the current suicidal ideation score (BSI-C, β = 9.189, t = 2.004, *p* = 0.048) among patients, but not among HC (β = 1.471, t = 0.825, *p* = 0.411), suggesting that gambling behavior has patient-specific predictive utility for suicidal symptoms.”

Page 19:

“Furthermore, linear regression showed that approach parameter can predict the current suicidal ideation score (β = 5.587, t = 2.890, *p* = 0.005) among patients, but not among HC (β = 0.874, t = 1.178, *p* = 0.241), suggesting that value-insensitive approach parameter has patient-specific predictive utility for suicidal symptoms.”

Page 21:

“Furthermore, linear regression showed that mood sensitivity to CR can predict the current suicidal ideation score (β = -28.706, t = -2.801, *p* = 0.006) among patients, but not among HC (β = -2.204, t = 0.528, *p* = 0.599), suggesting that mood sensitivity to CR has patient-specific predictive utility for suicidal symptoms.”

(3) The FDR correction for multiple comparisons mentioned briefly in lines 536-538 was not clear. Which analyses were included in the FDR correction? In particular, did the correlations between gambling rate and BSI-C/BSI-W survive such correction? Were there other correlations tested here (e.g., with the TAI score or ERQ-R and ERQ-S) that should be corrected for? Did the mediation model survive FDR correction? Was there a correction for other mediation models (e.g., with BSI-W as a predictor), or was this specific model hypothesized and pre-registered, and therefore no other models were considered? Did the differences in beta_gain across groups survive FDR when including comparisons of all other parameters across groups? Because the results were replicated in the online dataset, it is ok if they did not survive FDR in the patient dataset, but it is important to be clear about this in presenting the findings in the patient dataset.

Thank you for raising the important issue of multiple testing and for asking us to clarify exactly which tests were covered by the FDR procedure. In the clinical dataset we conducted a large number of inferential tests (χ^2^, t-tests, ANOVAs, regressions) spanning: (i) group differences in demographic/clinical characteristics; (ii) sanity checks (e.g., anxiety/depression questionnaires); (iii) primary hypotheses (e.g., group differences in risky behavior); (iv) model-based analyses (parameter checks and between-group contrasts); and (v) control/sensitivity analyses. Post-hoc t-tests were performed only when the three-group ANOVA was significant. This yielded >150 p-values. FDR was applied using all these p-values. Please see Supplementary Note 8.

(4) There is a lack of explicit mention when replication analyses differ from the analyses in the patient sample. For instance, the mediation model is different in the two samples: in the patient sample, it is only tested in S+ and S- groups, but not in healthy controls, and the model relates a dimensional measure of suicidal symptoms to gambling in the task, whereas in the online sample, the model includes all participants (including those who are presumably equivalent to healthy controls) and the predictor is a binary measure of S+ versus S- rather than the response to item 9 in the BDI. Indeed, some results did not replicate at all and this needs to be emphasized more as the lack of replication can be interpreted not only as "the link between mood sensitivity to CR and gambling behavior may be specifically observable in suicidal patients" (lines 582-585) - it may also be that this link is not truly there, and without a replication it needs to be interpreted with caution.

Thank you for these important comments. This study focused on cognitive and affective computational mechanisms underlying increased risky behavior in STB. Accordingly, we compared patients with STB (S^+^) with patients without STB (S^-^) and healthy controls (HC) to examine the effects of STB on risky behavior. Therefore, group comparison, instead of dimensional measure of suicidal symptoms by Beck Scale for Suicidal Ideation, can answer our research questions directly.

To enhance consistency between the clinical and replication datasets, we included all participants in each dataset when performing the mediation analysis. Given that S^-^ and HC did not differ in gambling behavior or the approach parameter in the clinical dataset, we merged these two groups. In the replication dataset, to mirror the S^+^ vs. S^-^ contrast used clinically, we categorized the general sample into S^+^ and S^-^ based on BDI item 9. The mediation results remained significant in both datasets (the clinical dataset: a×b = 0.321, 95% CI = [0.070, 0.549], *p* = 0.016; the replication dataset: a × b = 0.143, 95% CI = [0.016, 0.288], *p* = 0.031), suggesting that STB is associated with increased risk behavior via stronger approach motivation.

We also acknowledge the non-replication of the correlation between gambling behavior and mood sensitivity to certain rewards in the online sample. While this pattern might indicate that the link is specific to suicidal patients, it may also reflect sample-specific or unstable effects; thus, we now state this explicitly and interpret the finding with caution. Please see our revisions below:

Page 15:

“We next verified our results in an independent dataset, including the same task and BDI questionnaire in 747 general participants （500 females; age: 20.90±2.41）[46]. One item in BDI involves the measurement of STB. In item 9 of BDI, participants chose one option that describes them best: Option 1, “I don't have any thoughts of killing myself.”; Option 2, “I have thoughts of killing myself, but I would not carry them out.”; Option 3, “I would like to kill myself.”; Option 4, “I would kill myself if I had the chance.”. In line with the current definition of S^+^/S^-^ in the clinical dataset, we identified S^+^ group as choosing Option 2, 3, or 4, while participants selecting Option 1 were categorized as S^-^ group.”

Page 19:

“Given significant correlations between group, approach parameter, and gambling rate for gain trials (*ps* < 0.017), we further conducted a mediation analysis with the assumption of the mediating effect of approach motivation of suicidality on the risk behavior. Given that we aimed to test the effect of STB, with S^-^ and HC as controls, and given that S^-^ and HC did not differ in gambling behavior or in the approach parameter, we merged these two groups for the mediation analysis. Results supported our hypothesis (a×b = 0.321, 95% CI = [0.070, 0.549], *p* = 0.016; Figure 2C), confirming that suicidal thoughts and behavior increase risk behavior through stronger approach motivation.”

Page 26:

“However, we did not observe any significant correlation between mood sensitivity to CR and gambling behavior (*ps* > 0.389), which suggests that the link between mood sensitivity to CR and gambling behavior may be specifically observable in suicidal patients. Alternatively, this non-replicated result may also reflect sample-specific or unstable effects, which needs to be interpreted with caution.”

(5) In interpreting their results, the authors use terms such as "motivation" (line 594) or "risk attitude" (line 606) that are not clear. In particular, how was risk attitude operationalized in this task? Is a bias for risky rewards not indicative of risk attitude? I ask because the claim is that "we did not observe a difference in risk attitude per se between STB and controls". However, it seems that participants with STB chose the risky option more often, so why is there no difference in risk attitude between the groups?

Thank you for pointing out the ambiguity. In our manuscript, “motivation” and “risk attitude” are defined at the computational level. Following prior work with this task Rutledge et al., (2015, 2016), we decompose observed gambling into (i) value-dependent valuation parameters that capture risk attitude (e.g., risk aversion and loss aversion, which scale the subjective value of outcomes), and (ii) value-insensitive, valence-dependent biases that capture approach/avoidance motivation. Accordingly, a higher gambling rate does not imply a change in risk attitude per se: it can arise from an increased value-insensitive approach bias even when risk-attitude parameters are comparable between groups which is what we observe for S^+^ vs. controls. We have clarified this point in the computational modeling section.

Pages 12-13:

“Please note that a higher gambling rate does not imply a change in risk attitude per se: it can arise from an increased value-insensitive approach bias even when risk-attitude parameters are comparable between groups. Risk attitude is indeed conceptualized in economics as the curvature of the utility function (i.e., the subjective value) of the objective outcomes, with concave curves associated with risk aversion, and convex curves associated with risk seeking [54,56]. By contrast, the approach or avoidance bias apply to all the value. A possible interpretation of the approach bias is that participant approach the option with the highest possible gain (the lottery) in the gain frame; the avoidance bias would then reflect a tendency to systematically avoid the highest potential losses (the lottery) in the loss frame.”

**Reviewer #2 (Public review):**

Summary:

This article addresses a very pertinent question: what are the computational mechanisms underlying risky behaviour in patients who have attempted suicide? In particular, it is impressive how the authors find a broad behavioural effect whose mechanisms they can then explain and refine through computational modeling. This work is important because, currently, beyond previous suicide attempts, there has been a lack of predictive measures. This study is the first step towards that: understanding the cognition on a group level. This is before being able to include it in future predictive studies (based on the cross-sectional data, this study by itself cannot assess the predictive validity of the measure).

Strengths:

(1) Large sample size.

(2) Replication of their own findings.

(3) Well-controlled task with measures of behaviour and mood + precise and well-validated computational modeling.

Weaknesses:

I can't really see any major weakness, but I have a few questions:

(1) I can see from the parameter recovery that the parameters are very well identified. Is it surprising that this is the case, given how many parameters there are for 90 trials? Could the authors show cross-correlations? I.e., make a correlation matrix with all real parameters and all fitted parameters to show that not only the diagonal (i.e., same data is the scatter plots in S3) are high, but that the off-diagonals are low.

Thank you for raising these thoughtful concerns. The current task consisted of 90 choices and 36 mood ratings. There were 5 choice parameters and 4 mood parameters. The apparently strong identifiability is not unexpected, as 90 choice trials and 36 mood ratings are comparable to those in prior computational modeling literature (Blain & Rutledge, 2022).

As suggested, we computed cross-scorrelations between all generating (“true”) and recovered (“fitted”) parameters. The resulting matrix showed high diagonal (choice winning model: rs > 0.91; mood winning model: rs > 0.90) and low off-diagonal (choice winning model: abs(rs) < 0.63; mood winning model: abs(rs) > 0.40) correlations, further supporting parameter recovery. Please see Supplementary Pages 2-3.

“Parameter recovery: Figure S3 shows good parameter recovery for both choice and mood winning model (choice: rs > 0.91, *ps* < 0.001; intraclass coefficients > 0.78; mood: rs > 0.90, *ps* < 0.001; intraclass coefficients > 0.86). Moreover, we computed cross-correlations between all generating (“true”) and recovered (“fitted”) parameters. The resulting matrix showed high diagonal (choice winning model: rs > 0.91; mood winning model: rs > 0.90) and low off-diagonal (choice winning model: abs(rs) < 0.63; mood winning model: abs(rs) > 0.40) correlations, further supporting parameter recovery.”

Page 10：

“The numbers of choice trials and mood ratings were comparable to those in prior computational modeling studies [34,35].”

(2) Could the authors clarify the result in Figure 2B of a correlation between gambling rate and suicidal ideation score, is that a different result than they had before with the group main effect? I.e., is your analysis like this: gambling rate ~ suicide ideation + group assignment? (or a partial correlation)? I'm asking because BSI-C is also different between the groups. [same comment for later analyses, e.g. on approach parameter].

Thank you for pointing out the lack of clarity. We performed group difference analysis and correlation of suicidal ideation analysis, separately. We first performed group difference analysis to test our hypothesis of STB effects. We then conducted correlational analysis to further specify our findings.

(3) The authors correlate the impact of certain rewards on mood with the % gambling variable. Could there not be a more direct analysis by including mood directly in the choice model?

Thank you for this insightful suggestion. As suggested, we tried to integrate mood into choice models by adding mood bias component(s) in line with previous literature (Vinckier et al., 2018). The first model (mcM1) assumes that mood biases choice, building on cM3 (the winning choice model). cmM2 further separated the mood bias parameter into two components according to participants’ choices.

$$\displaystyle P_{\text {gamble}}=\frac{1-\beta_{\text {val}}}{1+e^{-\mu\left(U_{\text {gamble}}-U_{\text {certain}}\right)}}+\beta_{\text {val}} \text { if } \beta_{\text {val}} \geq 0$$

$$\displaystyle P_{\text {gamble}}=\frac{1+\beta_{\text {val}}}{1+e^{-\mu\left(U_{\text {gamble}}-U_{\text {certain}}\right)}} \text { if } \beta_{\text {val}}\lt0$$

$$\displaystyle \beta_{\text {val }}\left\{\begin{array}{cl}\beta_{\text {gain }}, & \text { gain trials }, \\ \beta_{\text {loss }}, & \text { loss trials } .\end{array}\right.$$

However, model comparison using BIC supported cM3 (Table S6), that is, without consideration of mood in choice modeling. This can be due to the lack of block design in our experimental design unlike e.g., Vinckier et al., (2018) and Eldar & Niv, (2015). Please see Supplementary Note 6.

(4) In the large online sample, you split all participants into S+ and S-. I would have imagined that instead, you would do analyses that control for other clinical traits. Or, for example, you have in the S- group only participants who also have high depression scores, but low suicide items.

Thank you for this insightful suggestion. Following prior suicide-related literature (Tsypes et al., 2024), we controlled for depression by including them as covariates. Note that depression scores were derived from our established bifactor model (Wang et al., 2025), which decomposed depression from the anxiety. These results remained largely significant (*ps* ≤ 0.050), except a marginally significant effect of group on gambling behavior (*p* = 0.059). Despite a trend, this effect with covariates of depression-related questionnaires is strong in our clinical cohort (*p* = 0.024; Table S8). This suggests that the link between suicidality and risky behavior persists above and beyond general depressive symptoms.

Please see our clarifications below:

Page 26:

“After controlling for depression severity using our established bifactor model (see ref 60 for details), these results remained significant (*ps* ≤ 0.050), except a marginally significant effect of group on gambling behavior (*p* = 0.059). Despite a trend, this effect with covariates of depression-related questionnaires is strong in our clinical cohort (*p* = 0.024; Table S8). This suggests that the link between suicidality and risky behavior persists above and beyond general depressive symptoms.”

**Reviewer #3 (Public review):**

This manuscript investigates computational mechanisms underlying increased risk-taking behavior in adolescent patients with suicidal thoughts and behaviors. Using a well-established gambling task that incorporates momentary mood ratings and previously established computational modeling approaches, the authors identify particular aspects of choice behavior (which they term approach bias) and mood responsivity (to certain rewards) that differ as a function of suicidality. The authors replicate their findings on both clinical and large-scale non-clinical samples.

(1) The main problem, however, is that the results do not seem to support a specific conclusion with regard to suicidality. The S+ and S- groups differ substantially in the severity of symptoms, as can be seen by all symptom questionnaires and the baseline and mean mood, where S- is closer to HC than it is to S+. The main analyses control for illness duration and medication but not for symptom severity. The supplementary analysis in Figure S11 is insufficient as it mistakes the absence of evidence (i.e., p > 0.05) for evidence of absence. Therefore, the results do not adequately deconfound suicidality from general symptom severity.

Thank you for this important comment. Based on clinical interviews, we included patients with and without suicidality (S^+^ and S^-^ groups). However, in line with suicidal-related literature (e.g., Tsypes et al., 2024), two groups also differed substantially in the severity of symptoms (see Table 1). To address the request for evidence on specificity to suicidality beyond general symptom severity, we performed separate linear regressions to explain in gambling behaviour, value-insensitive approach parameter (β_gain_), and mood sensitivity to certain rewards (β_CR_) with group as a predictor (1 for S^+^ group and 0 for S^-^ group) and scores for anxiety and depression as covariates. Results remained significant after controlling anxiety and depression (*ps* < 0.027; Table S8). Given high correlations among anxiety and depression questionnaires (rs > 0.753, *ps* < 0.001), we performed Principal Components Analysis (PCA) on the clinical questionnaire to extract the orthogonal components, where each component explained 86.95%, 7.09%, 3.27%, and 2.68% variance, respectively. We then performed linear regressions using these components as covariates to control for anxiety and depression. Our main results remained significant (*ps* < 0.027; Table S9). We believe that these analyses provide evidence that the main effects on gambling and on mood were specific to suicide.

As pointed out, these “absence of evidence” cannot provide insights of “evidence of absence”. Although we median-split patients by the scores of general symptoms (e.g., depression and anxiety-related questionnaires) and verified no significant differences in these severities (Figure S11), we additionally conducted Bayesian statistics in gambling behavior, value-insensitive approach parameter, and mood sensitivity to certain rewards. BF_01_ is a Bayes factor comparing the null model (M_0_) to the alternative model (M_1_), where M_0_ assumes no group difference. BF_01_ > 1 indicates that evidence favors M_0_. As can be seen in Table S7, most results supported null hypothesis, suggesting that general symptoms of anxiety and depression overall did not influence our main results. Overall, we believe that these analyses provide compelling evidence for the specificity of the effect to suicide, above and beyond depression and anxiety.

Please see Table S7, S8 &S9 and our revisions below.

Page 17:

“Within patients, this group effect on gambling rate remained significant after controlling for sex, illness duration, family history, diagnosis, and various medications use (*ps* < 0.05), as well as general symptoms (e.g., depression and anxiety; *p* = 0.024; also see Figure S11, Table S7 and Table S8). Given high correlations among anxiety and depression questionnaires (rs > 0.753, *ps* < 0.001), we performed Principal Components Analysis (PCA) to extract main components, where each component explained 86.95%, 7.09%, 3.27%, and 2.68% variance, respectively. To further control for anxiety and depression, linear regression using these components as covariates revealed that the group effect on gambling rate remained significant (*p* = 0.024; Table S9).”

Pages 18-19:

“Within patients, this group effect on the approach parameter remained significant after controlling for sex, illness duration, family history, diagnosis, and various medications use (*ps* < 0.05), as well as general symptoms (e.g., depression and anxiety; *p* = 0.027; also see Figure S11, Table S7 and Table S8). Linear regression using PCA components as covariates revealed that the group effect on approach parameter remained significant (*p* = 0.027; Table S9).”

Page 21:

“Within patients, this group effect on βCR remained significant after controlling for gambling rate, earnings, mood-related outcome effect, mood drift effect, sex, illness duration, family history, diagnosis, and various medications use (ps < 0.032), as well as general symptoms (e.g., depression and anxiety; p = 0.001; also see Figure S11, Table S7 and Table S8). Linear regression using PCA components as covariates revealed that the group effect on this mood parameter remained significant (p = 0.001; Table S9).”

(2) The second main issue is that the relationship between an increased approach bias and decreased mood response to CR is conceptually unclear. In this respect, it would be natural to test whether mood responses influence subsequent gambling choices. This could be done either within the model by having mood moderate the approach bias or outside the model using model-agnostic analyses.

Thank you for this important suggestion. As suggested, one interesting question was whether mood responses influence subsequent gambling choices and how to model them. First, we median-split mood responses (except the final rating) to compare gambling rate. Results showed a trend for less gambling rate in higher mood (t = -1.971, *p* = 0.050). However, there was no significant group difference (F = 0.680, *p* = 0.507). Second, with the assumption that mood biases choice, we constructed mcM1 based on cM3 (the winning choice model). Based on our finding of the negative correlation between mood sensitivity to certain rewards and gambling rate in S^+^, we separated β_Mood_ parameter into β_Mood-CR_ and β_Mood-GR_ (cmM2). Model comparison using BIC supported cM3 (Table S6), that is, without consideration of mood in choice modeling. This can be due to the lack of block design in our experimental design unlike e.g., Vinckier et al., (2018) and Eldar & Niv, (2015). Please see Supplementary Note 6.

(3) Additionally, there is a conceptual inconsistency between the choice and mood findings that partly results from the analytic strategy. The approach bias is implemented in choice as a categorical value-independent effect, whereas the mood responses always scale linearly with the magnitude of outcomes. One way to make the models more conceptually related would be to include a categorical value-independent mood response to choosing to gamble/not to gamble.

We apology for the unclear statement. The approach bias is implemented in choice as a continuous value-independent effect, ranging from -1 to 1.

$P_{\text {gamble}}=\frac{1-\beta_{\text {val}}}{1+e^{-\mu\left(U_{\text {gamble}}-U_{\text {certain}}\right)}}+\beta_{\text {val}} \text { if } \beta_{\text {val}} \geq 0$

$P_{\text {gamble}}=\frac{1+\beta_{\text {val}}}{1+e^{-\mu\left(U_{\text {gamble}}-U_{\text {certain}}\right)}} \text { if } \beta_{\text {val}}\lt0$

$\beta_{\text {val }}\left\{\begin{array}{cl} \beta_{\text {gain }}, & \text { gain trials }, \\ \beta_{\text {loss }}, & \text { loss trials. } \end{array}\right.$

It was true that the mood responses always scale with the magnitude of outcomes, since mood ratings were request after the outcomes. Therefore, mood parameters and the approach bias were both continuous.

We also attempted to integrate mood into choice modelling. See Response 2 for Reviewer 3 for details.

(4) The manuscript requires editing to improve clarity and precision. The use of terms such as "mood" and "approach motivation" is often inaccurate or not sufficiently specific. There are also many grammatical errors throughout the text.

Thank you for this important suggestion. We have now explained motivation and mood in the Introduction section and the computational modeling section. Please see our clarifications below:

Pages 3-4:

“A growing literature indeed shows that risky behavior can be far better explained after adding value-insensitive approach and avoidance components to prospect theory [18,19], that is by including a decision bias in favor of the highest gain (approach) and another decision bias against the lowest loss (avoidance), above and beyond options value difference. This class of models highlights the important role of value-insensitive motivational components in decision making in addition to risk attitude-driven valuation (e.g., loss/risk aversion) [20].”

Page 5:

“Although mood is thought to persist for hours, days, or even weeks [30–33], momentary mood, measured over the timescale in the laboratory setting, represents the accumulation of the impact of multiple events at the scale of minutes [30,32,34–38]. Momentary mood external validity is demonstrated e.g., through its association with depression symptoms [37]. Mood is different from emotions, which reflect immediate affective reactivity and is more transient (e.g., from surprise to fear) [31–33,39].”

We have corrected grammatical errors throughout the manuscript.

(5) Claims of clinical relevance should be toned down, given that the findings are based on noisy parameter estimates whose clinical utility for the treatment of an individual patient is doubtful at best.

Thank you for this comment. We agree that we did not evaluate the noise in our estimate e.g., by assessing the test-retest reliability on the task parameters, which is outside the scope of the study, and it is indeed possible that parameter estimate is somehow noisy. Therefore, we tone down the clinical relevance of our results. Please see our revision below:

Page 32:

“Next, we did not evaluate the noise in our estimate e.g., by assessing the test-retest reliability on the task parameters and it is indeed possible that parameter estimate is somehow noisy.”

**Recommendations for the authors:**

**Reviewer #1 (Recommendations for the authors):**

(1) Title: I believe "aberrant mood dynamics" is both too general and overstating the results of this study, which did not measure mood dynamics longitudinally. "Aberrant" is also overly pathologizing. I would suggest sticking more directly to the results, for instance, "Insensitivity of momentary mood to non-risky rewards in adolescent suicidal patients".

Thank you for this suggestion. We have now corrected it.

(2) Abstract: in line 61, "Our study uncovers the cognitive and affective mechanisms" suggests that these are the only ones, and you uncovered them. Of course, there could be more mechanisms contributing to risk behavior in STB, so I would suggest removing the word "the" or adding "one of the".

Thank you for this suggestion. We have now corrected it.

(3) One major weakness of this study is that suicidal thoughts and behaviors were not assessed via a clinical instrument such as the Columbia Suicide Severity Rating Scale - this should be mentioned upfront.

Thank you for this comment. According to medical records and information from family and friends by the researcher and psychiatrists, patients with suicidal thoughts and behaviors were categorized as suicidal group (S^+^), while patients without suicidal thoughts and behaviors were identified as control group (S^-^). Note that medical records and information were recorded from clinical interviews where the psychiatrists were vigilant for signs of suicidal ideation and inquired about suicidal-related thoughts and behaviors from both the patients and their families. Therefore, the current group operation was possibly comparable to Columbia Suicide Severity Rating Scale.

(4) Table 1: female/male are sex, not gender (gender is man/woman/transgender/non-binary).

Thank you for this suggestion. We have now corrected it.

(5) Equation 1: It would be good to clarify what happens in gain-only or loss-only trials (the other value is then 0, but this can be clarified as it is not technically a loss or a gain).

Thank you for this suggestion. We have now corrected it. Please see below for our revision:

Page 12:

“Please note that V_*gain*_ is 0 in gain trials and V_*loss*_ is 0 in loss trials.”

(6) Figure 1E: The model prediction is not informative here. Given the linear regression model, there is no other option except that the mean prediction would overlap with the mean empirical measurement (unless the model was specified incorrectly). The same is true in Figure 2A.

Thank you for this suggestion. We have now removed plots for model prediction.

(7) Figure 1G: There was no analysis of the differences between groups in terms of earnings, given that the ANOVA was not significant. Still, if the claim is that risky behavior is sometimes suboptimal in this task, it would be good to show that there is a correlation between, say, symptoms of STB across groups and (1) risky behavior and (2) earnings.

Thank you for this insightful comment. In the patient cohort, risky behavior (gambling rate)—but not earnings predicted the current suicidal ideation score (BSI-C, β = 9.189, t = 2.004, *p* = 0.048; earnings, β = 0.001, t = 0.582, *p* = 0.562). The lack of association for earnings is consistent with the task design, in which there is no stable optimal policy and payouts are only a coarse proxy for decision quality. Future work in learning paradigms, where optimality is well defined, may be better suited to test earning-based links to STB. We have clarified this point below:

Page 32:

“Second, although we assumed that increased risky behavior in STB was suboptimal, the current task was not suited to test this, given the task design of random feedback for gambling option. Future work in learning paradigms, where optimality is well defined, may be better suited to test earnings-based links to STB.”

(8) Line 290: "beta_gain: -1-1" is unclear. I believe you meant beta_gain \in [-1,1].

Thank you for this suggestion. We have now corrected it to make it clear.

(9) The gain and loss biases are modeled as minimum and maximum probabilities for choosing the gamble. This is a legitimate choice for value-agnostic biases, but it is not the traditional choice (as far as I know). I wonder if the same results would hold with the more traditional formulation of the bias as an added constant to the utility of the gamble, i.e., p(gamble) = 1/(1+ exp(-mu(U_gamble + beta_gain - U_certain))). I believe in this case, you would also not have to specify different equations for positive or negative biases, or to limit the bias to the range of [-1,1] (indeed, the bias would be in reward-equivalent units).

Thank you for this suggestion. The winning choice model we used here was consistent with previous literature (Rutledge et al., 2015 & 2016), which decomposed the decision process into risk-attitude-driven valuation (e.g., loss and risk aversion) and value-insensitive motivational components. These approach/avoidance parameters are a decision bias in favor of the highest gain (approach) and another decision bias against the lowest loss (avoidance), above and beyond options value difference.

As suggested, we also compared the traditional bias choice model. Model comparison did not support this. Please see Supplementary Page 4.

(10) Also, for equations 5-8, it seems that 5-6 are identical to 7-8 except for the use of beta_gain versus beta_loss. You might want to consider simplifying by putting beta in the equations and specifying in the text that, depending on the trial type (loss or gain), the relevant beta is used.

Thank you for this suggestion. We have now simplified it. Please see our revision below:

$P_{\text {gamble}}=\frac{1-\beta_{\text {val}}}{1+e^{-\mu\left(U_{\text {gamble}}-U_{\text {certain}}\right)}}+\beta_{\text {val}} \text { if } \beta_{\text {val}} \geq 0$

$P_{\text {gamble}}=\frac{1+\beta_{\text {val}}}{1+e^{-\mu\left(U_{\text {gamble}}-U_{\text {certain}}\right)}} \text { if } \beta_{\text {val}}\lt0$

$\beta_{\text {val }}\left\{\begin{array}{cl} \beta_{\text {gain }}, & \text { gain trials }, \\ \beta_{\text {loss }}, & \text { loss trials. } \end{array}\right.$

(11) It is not clear what equations are applied to mixed trials in cM3.

Sorry for the confusion. We have now clarified this point.

Page 12:

“Approach/avoidance parameters are not applied to in mixed trials.”

(12) Model comparison: the mood models are nested within each other (e.g., mM3 can be derived from mM1 by setting beta_EV = beta_RPE). In this case, model comparison can use the likelihood ratio test instead of BIC, which can be too conservative (and therefore does not support the extra beta parameter for RPE, different from previous results in the literature). I wonder if a likelihood ratio test would lead to results more in line with previous findings with this task?

Thanks for this suggestion. We agree that mM1 (CR+EV+RPE) and mM3 (CR+GR) are nested. However, our model space also included unnested models, such as mM5 (CR+GR_better_+GR_worse_). Therefore, it was not reasonable in our model space to use likelihood ratio tests.

(13) Line 346: The replication sample is described as "healthy participants," however, their health (or mental health) status was not assessed, and they may as well have mental health concerns. I would suggest calling this a general sample or an undifferentiated sample - but not a healthy sample.

Sorry for the confusion. We have now corrected this phrase.

(14) Line 363: "in addition to the replication of previous findings in the validation dataset" is unclear. Are those tests not two-tailed?

Sorry for the unclear statement. In the replication analyses, we used one-tailed t-tests because the direction of the effect was revealed on the clinical dataset. Please see our clarification below:

Page 15:

“For the replication of previous findings in the validation dataset, we used one-tailed tests in line with our clinically motivated directional hypothesis.”

(15) Line 372: "validating our group manipulation" - the presented work does not have a manipulation. Maybe you meant "validating our grouping of participants"?

Thank you for this suggestion. We have now corrected it to make it clear.

(16) Figure 2B: It is not clear how the data were binned for illustration purposes only, and why this binning is necessary (I have not seen it in other papers) - presenting the data from each subject and the correlation line with error margins (as is done here) should be sufficient.

Thank you for flagging this. For illustration only, we binned the data proportional to group sizes: in the patient sample (S^-^ *n* = 25; S^+^ *n* = 58; ≈1:2), we displayed 3 bins for S^-^ and 6 bins for S^+^. We agree that binning is not necessary; all statistics were computed on raw, unbinned data. The binned panel was included solely for visualization, consistent with our prior work (Blain et al., 2023).

(17) Table 2: delta BIC should be presented per subject (that is, divided by the number of subjects in each group), as the groups are of different sizes, so as presented now, the columns are not comparable across groups.

Thank you for the helpful suggestion. Our goal in Table 2 is not to compare ΔBIC magnitudes across groups, but to identify the winning model within each group. The ΔBICs are aggregated at the group level solely to rank models for that group. Dividing by the number of participants would rescale each group’s column by a constant and would therefore not affect the within-group ranking or the conclusion that cM3 is the best model in all groups. For this reason, we retain the current presentation and interpret each column within group rather than across groups.

(18) Line 640 - the effect of expectations and prediction errors on mood was not only shown in healthy people, but also in people with depression (Rutledge et al., 2007, https://pubmed.ncbi.nlm.nih.gov/28678984/)

Thank you for this comment. Indeed, Rutledge et al., (2017) showed evidence for CR+EV+RPE mood model in adult people with depression. However, our study recruited adolescents with depression or anxiety, given that adolescent period might provide a developmental window for opportunities for early intervention of suicidality. Therefore, it is also possible that the current winning model was specific to adolescents. Please see our clarifications below:

Page 28:

“It is also possible that the current winning model was specific to adolescents. Given that Rutledge et al., (2017) supported the “CR-EV-RPE model” in adults with depression, our study with adolescent populations may suggest a developmental change for mood sensitivities.”

(19) Supplemental material: Is the R2 section about R-squared? Perhaps you can use superscript on the 2 to make that clearer? For Figure S2, how was model recovery determined? Should I interpret the confusion matrix as suggesting that the winning model for each and every simulated subject was the generating model, or was the winning model determined for the whole simulated population in each of the 100 simulations? Traditionally, confusion matrices use the former measure, but the results of 100% recoverability make me suspect the latter was used here. In Figure S3, should we not be looking at simulated parameters and recovered parameters? What are "real parameters" here?

Thank you for these important comments. We now consistently denote the coefficient of determination as R^2^ (with a superscript 2) throughout the manuscript and Supplementary Materials.

For the model recovery analysis in Figure S2, we have clarified that the confusion matrix is computed at the population level. Specifically, for each of the 100 simulations we generated a full dataset under each candidate model, fit all models to that dataset, and selected the winning model based on group-level model evidence (BIC). Each cell in the confusion matrix therefore reflects the proportion of simulations in which model *j* was selected as the best-fitting model when the data were generated by model *i*. This operation was reasonable because the decision of the winning model is made on the population-level dataset rather than on individual subjects.

In Figure S3, the term “real parameters” referred to the parameters used to generate the simulated data. To avoid confusion, we now relabel these as “simulated (generating) parameters” and explicitly describe the figure as showing the relationship between simulated (generating) parameters and recovered parameters. Please see Supplementary Pages 2-3:

“Model recovery： We generated 100 simulated datasets for each model (3 choice models and 8 mood models) using the fitted parameters of each model as the ground truth. Each dataset contained 201 trials and included 3 (or 8) sets of simulated data corresponding to the respective models. For each simulated dataset, we then fit all models and determined the winning model at the population level based on group-level BIC, yielding a confusion matrix in which each entry represents the proportion of simulations in which model *j* was selected as the best-fitting model when the data were generated by model *i*. As shown in Figure S2, all models are highly identifiable, indicating excellent recovery performance for both the choice and mood models.”

“Parameter recovery: Figure S3 shows good parameter recovery for both choice and mood winning model (choice: rs > 0.91, *ps* < 0.001; intraclass coefficients > 0.78; mood: rs > 0.90, *ps* < 0.001; intraclass coefficients > 0.86). Moreover, we computed cross-correlations between all generating (“generating”) and recovered (“fitted”) parameters. The resulting matrix showed high diagonal (choice winning model: rs > 0.91; mood winning model: rs > 0.90) and low off-diagonal (choice winning model: abs(rs) < 0.63; mood winning model: abs(rs) > 0.40) correlations, further supporting parameter recovery.”

Typos:

(1) Line 90: original → originate

(2) Line 596-598 - the same phrase is repeated twice.

(3) Line 616: on the other word → hand.

Sorry for the mistakes. We have now corrected them throughout the manuscript.

**Reviewer #2 (Recommendations for the authors):**

For people unfamiliar with interpersonal theory or motivational-volitional model, or three-step theory (lines 105-106), could you briefly explain the key idea of mood and suicide before going to the decision-making tasks? And from this, maybe motivate the predictions in your task? In particular, in the abstract and introduction, the phrasing could be a bit more concise and simpler. In the abstract, sentences were sometimes quite long. In the introduction, some paragraphs are somewhat repetitive. In the discussion, there were some typos.

Thank you for these suggestions. We have now explained the key idea of mood and suicide before going to the decision-making tasks in the introduction, which can be seen below:

Pages 4-5:

“Contemporary theories of suicide converge on the idea that STB is initially caused by low mood experience. The interpersonal theory of suicide proposes that suicidal desire arises when people simultaneously feel socially disconnected (“thwarted belongingness”) and like a burden on others (“perceived burdensomeness”), experiences that are tightly linked to chronically low mood [25]. The motivational–volitional model [26] and the three-step theory [27,28] similarly emphasize that when negative mood and feelings of defeat or entrapment are experienced as inescapable, they can give rise to suicidal ideation, and that the progression from ideation to suicide attempts depends on additional factors such as reduced fear of death, increased pain tolerance, and a tendency to act impulsively under intense affect. Some official organizations, e.g., National Institute of Mental Health, have also listed mood problems as warning signals [8]. Interestingly, within the framework of decision making under uncertainty, gambling on lotteries with a revealed outcome has been found to induce high mood variance [29], providing an opportunity to assess the relationship between deficient mood and increased gambling decisions in STB.”

We have also refined the wording and corrected typos throughout the manuscript.

**Reviewer #3 (Recommendations for the authors):**

(1) Since many readers might only read the abstract, it is important that it is both informative and accurate. I have two suggestions in this respect. First, for the abstract to be more informative, it may be helpful to indicate already there that these are value-insensitive approach-avoidance parameters, in the sense that they favor/disfavor the gamble regardless of the potential outcomes' magnitude or probability. This issue is also present throughout the text, where the phrases "approach and avoidance motivation" are referred to as if they have established and precise computational definitions. In my view, these terms could just as easily be interpreted as parameters that multiply the value of potential gains or losses, which is not what the authors mean. It would be helpful to clarify this terminology.

Thank you for these suggestions. In line with previous literature (Rutledge et al., 2015 & 2016), approach and avoidance motivation are indeed defined at the computational level, referring to a decision bias in favor of the highest gain (approach) and another decision bias against the lowest loss (avoidance), above and beyond options value difference. We have cited these papers in the manuscript. We also make it clear to further clarify approach and avoidance parameters in the abstract and introduction. Please see our revisions below:

Page 2 (Abstract):

“Using a prospect theory model enhanced with value-insensitive approach-avoidance parameters revealed that this rise in risky behavior resulted only from a heightened approach parameter in S^+^.”

“Altogether, model-based choice data analysis indicated dysfunction in the approach system in S^+^, leading to greater propensity for gambling in the gain domain regardless of the lottery expected value.”

Page 3 (Introduction):

“A growing literature indeed shows that risky behavior can be far better explained after adding value-insensitive approach and avoidance components to prospect theory [18,19], that is by including a decision bias in favor of the highest gain (approach) and another decision bias against the lowest loss (avoidance), above and beyond options value difference. This class of models highlights the important role of value-insensitive motivational components in decision making in addition to risk attitude-driven valuation (e.g., loss/risk aversion) [20].”

(2) The statement "our study uncovers the cognitive and affective mechanisms contributing to increased risk behavior in STB" is overstating the findings, as the study may have uncovered some contributing mechanisms, but likely not all of them. Removing the word "the" would fix this issue.

Thank you for this suggestion. We have now corrected it.

(3) Since mood is typically defined as lasting hours, it's inappropriate to refer to ratings that only reflect the last few trials as self-reports of mood. To be sure, I view the distinction between emotions and moods as quantitative, not qualitative, so I do not think there is a problem studying the former to understand the latter, but to avoid confusion, the terminology should follow common usage.

Thank you for this suggestion. We follow previous work and operational definitions regarding mood (Rutledge et al., 2014, Eldar & Niv, 2015, Vinckier et al., 2018). Emotion is usually a very brief response to a specific stimulus (Emanuel & Eldar, 2023), e.g., leading to rapid changes like surprise then fear. In contrast, mood is defined as a diffuse state that is not specific to one stimulus. Here, we operationally and computationally define mood as an affective state reflecting the recent history of safe and gamble outcomes. We now clarify that point in the main text. Please see our revision below:

Page 5:

“Although mood is thought to persist for hours, days, or even weeks [30–33], momentary mood, measured over the timescale in the laboratory setting, represents the accumulation of the impact of multiple events at the scale of minutes [30,32,34–38]. Momentary mood external validity is demonstrated e.g., through its association with depression symptoms [37]. Mood is different from emotions, which reflect immediate affective reactivity and is more transient (e.g. from surprise to fear) [31–33,39].”

(4) Line 78: The phrases "increase in risk attitude", "decrease in loss attitude", and "decrease in value-independent choice biases" are unclear to me in terms of their directionality. An attitude might be avoidant or embracing. If it is the former then increasing it would decrease risk-taking.

Thank you for pointing out the ambiguity. We have now corrected them throughout the manuscript. Please see our revision below:

Page 4:

“We therefore hypothesized that heightened approach motivation, or weakened avoidance motivation, would account for increased risk behavior in STB.”

(5) Line 125: I was not sure why one would expect the mood response to gamble-related quantities (EV and RPE) to be lower in STB and not higher.

Sorry for the typo. We hypothesized that mood would respond more strongly to gambling-related quantities expected value (EV) and reward prediction error (RPE)—in adolescents with STB than in controls, given prior evidence that STB is associated with greater risk-taking.

(6) The text could use proofreading, as there are many typos. These are from the first 100 lines alone:

(a) Abstract: regardless the lotteries -> regardless of the lotteries'.

(b) Line 78: it remains whether.

(c) Line 80: can each -> each can.

(d) Line 90: may original from.

Sorry for the mistakes. We have now corrected them throughout the manuscript.

(7) The rationale for focusing on the S+ group for mood model comparison is incorrect. The purpose is to identify parameters that vary as a function of suicidality, and for that, the S- group is just as important.

Thank you for this comment. We agree that the S^-^ group is as important as the S^+^ group. A direct comparison was complicated because the winning mood models differed (S^+^: mM3; S^-^: mM5; Table 3). To ensure comparability, we checked results from both model specifications (mM3 and mM5). The conclusions were convergent: mood sensitivity to certain rewards (CR) was lower in S^+^ than in S^-^ (see Fig. 3 for mM3 and Fig. S8 for mM5).

(8) There appears to be a contradiction between the inclusion criteria, which include having experienced suicidal thoughts and behaviors, and the definition of the S- group as not having suicidality.

Thank you for pointing out this mistake. The corrected version of inclusion criteria can be seen on Page 7:

“Patients were included if they met the following criteria: (1) both the researcher and psychiatrists agreed on their group classification; (2) they had a current diagnosis of major depressive disorder (MDD; unipolar depression), generalized anxiety disorder (GAD), or bipolar disorder with depressive episodes (BD), confirmed by two experienced psychiatrists using the Structured Clinical Interview for DSM-IV-TR-Patient Edition (SCID-P, 2/2001 revision; see Supplementary Note 1 for details)；(3) they were between 10 and 19 years of age; (4) they had no organic brain disorders, intellectual disability, or head trauma; (5) they had no history of substance abuse; (6) they had no experience of electroconvulsive therapy.”

(9) It would be helpful to specify whether mood modeling was based on objective or subjective values, and why.

Thank you for this helpful suggestion. We have now clarified whether mood modeling was based on objective or subjective values, and why. Specifically, we constructed two model families: one in which mood was driven by objective monetary outcomes (objective values) and one in which mood was driven by subjective values derived from each participant’s fitted choice model (subjective values). We then used the VBA_groupBMC function in the VBA toolbox to perform family-wise model comparison, with 8 candidate mood models within each family. Consistent with previous literature, the objective-value family provided a clearly superior fit to the data (exceedance probability, EP = 1.000). Based on this result and for parsimony, we report and interpret the mood modeling results from the objective-value family in the main text. We have clarified this point in Supplementary Note 9.
